# Glutamine modulates stress granule formation in cancer cells through core RNA-binding proteins

**DOI:** 10.1242/jcs.263679

**Published:** 2025-06-06

**Authors:** Gabriel P. Faber, Gilad Gross, Oz Mualem, Matan Y. Avivi, Hiba Waldman Ben-Asher, Orly Yaron, Noa Kinor, Orit Shefi, Rakefet Ben-Yishay, Dana Ishay-Ronen, Yaron Shav-Tal

**Affiliations:** ^1^ The Mina & Everard Goodman Faculty of Life Sciences, Institute of Nanotechnology, Bar-Ilan University, Ramat Gan 5290002, Israel; ^2^ Faculty of Engineering, Institute of Nanotechnology, Bar-Ilan University, 5290002 Ramat Gan, Israel; ^3^ Oncology Institute, Chaim Sheba Medical Center, Tel-Hashomer, 5262100 Ramat Gan, Israel

**Keywords:** RNA FISH, Stress granules, Glutamine, Myc

## Abstract

Cytoplasmic stress granules (SGs) induced by various stresses have been linked to cancer and other disorders. Which active energy pathways are required for SG formation remains unclear. We used nutrient deprivation to show that glutamine is the sole amino acid source governing whether cancer cells form SGs. Metabolic profiling revealed the essential functions of glutamine and glucose in SG formation under limiting metabolic conditions. Providing glutamine during metabolic stress restored ATP levels in cancer cells and revived many essential gene expression patterns. *MYC*, a known regulator of the shift between glucose and glutamine metabolism, showed increased expression as cells moved to glutamine uptake. Inhibition of MYC prevented SG formation even with glutamine present and increased cell death after arsenite exposure. The RNA-binding proteins G3BP1 and G3BP2 (collectively G3BP1/2) were required for glutamine utilization, with G3BP1/2-knockout cells displaying a heavier reliance on glucose, yielding reduced cell survival and an inability to properly utilize glutamine. Altogether, we show that cancer cells require glutamine for SG formation under nutrient deprivation, and its absence reduces cell survival, lowering ATP levels below an energy threshold required for SG formation.

## Teaser

**Highlighted Article:** Cells require both glutamine and glucose to form SGs and to maintain normal cell function during nutrient stress.

## INTRODUCTION

Cytoplasmic stress granules (SGs) are membraneless condensates that harbor translationally stalled mRNAs along with a wide variety of RNA-binding proteins (Anderson and Kedersha, 2006; Protter and Parker, 2016; Tian et al., 2020) and have been linked to a variety of diseases (Wang et al., 2022a). Included among the proteins found in SGs are those that are part of machinery essential for returning transcripts to polysomes for active translation, such as initiation factors and ribosomal subunits (Campos-Melo et al., 2021). Although other cytoplasmic bodies, such as processing bodies (PBs), are microscopically visible under normal conditions, SGs only form upon the induction of certain cellular stresses (Erickson and Lykke-Andersen, 2011; Riggs et al., 2020). The stressors induce SG formation through the integrated stress response (ISR) pathway, which can activate four different kinases, all of which phosphorylate eIF2α, thereby stalling translation (Ivanov et al., 2019). Altogether, SGs represent a cohesive class of granules, but their contents and function are dictated by the stress and environment in which they were induced (Aguilera-Gomez et al., 2017).

SGs form through liquid–liquid phase separation (LLPS), a process driven by protein–protein, RNA–protein, and RNA–RNA interactions (Angel et al., 2024; Protter and Parker, 2016; Riggs et al., 2020). The resultant multivalent structure of the condensates has distinct stages of assembly and contains many proteins. Central among the proteins is the RNA-binding protein G3BP1, a modulator of the core of the SG structure (Hofmann et al., 2021; Jain et al., 2016; Wheeler et al., 2016). Microtubules (MTs) are also thought to play a role in the dynamics of SG formation, with studies showing that disruption of the microtubule network completely abolishes SG formation (Ivanov et al., 2003; Kolobova et al., 2009; Kwon et al., 2007). It has further been suggested that the interactions of proteins with free tubulin negate their ability to form fully mature SGs (Chernov et al., 2009). Other studies have found that there is an increased number of smaller, more dispersed granules featuring impaired fusion and slower movement due to a lack of anchoring to MTs and loss of their retrograde movement when MTs are disrupted (Fujimura et al., 2009; Nadezhdina et al., 2010). The accepted model is that SGs slide along MTs to fuse and form larger granules, and in the absence of MTs, small granules nucleate via hindered diffusion (Chernov et al., 2009; Maucuer et al., 2018). Taken together, SGs are formed, maintained and recruit proteins through a complex network of interactions, some of which are MT dependent (Fujimura et al., 2009).

Other studies have examined the energy dependence of the SG assembly pathway. Super-resolution imaging has been used to uncover the SG core and to highlight ATP-dependent remodelers as conserved components of SGs (Aguilera-Gomez et al., 2017). It has been further demonstrated that ATP plays a role in SG assembly and dynamics in some situations, although the dynamic exchange of factors entering and exiting the granules is still observed upon ATP depletion (Jain et al., 2016). Another study has shown how the use of an ATP mimetic inhibitor interferes with SG maturation rather than with their formation (Eum et al., 2020). Chronic glucose starvation has also been reported to induce SG formation (Reineke et al., 2018), and SGs that form after severe glucose deprivation show different shapes and compositions compared to canonical SGs (Wang et al., 2022b). A theoretical model has suggested that ATP is continuously hydrolyzed to prevent SG formation, as a sort of energy ‘insurance plan’, such that only a little additional ATP is required to initiate SG assembly when cells are exposed to stress, when energy is expected to be in short supply and the timing of stress onset is unpredictable (Wurtz and Lee, 2018).

The role of amino acids in SG formation is still little understood. One study has shown that restraining glutamine availability limits SG formation in KRAS-driven pancreatic cancer, which is thought to limit chemoresistance (Mukhopadhyay et al., 2020). Cancer cells are known to be ‘addicted’ to glutamine, with the oncoprotein MYC ensuring the uptake and consumption of high amounts of glutamine (Altman et al., 2016; Hensley et al., 2013; Wise et al., 2008). In the present study, we looked for stress conditions that would abolish the formation of SGs and reduce cancer cell survival. We examined how depriving cancer cells of energy by interfering with their glutamine and glucose metabolic pathways, and the ATP they produce, would affect SG formation and cell survival. We found that when we combined amino acid (AA) starvation with a chemotherapy used to disrupt the cytoskeleton of cancer cells, we could prevent SG formation entirely. AA starvation had a pronounced effect on the amount of SG formation, whereas the absence of MTs changed SG morphology. Interestingly, when a single AA, glutamine, was returned to the cell culture during AA starvation, SGs returned to almost full abundance. AA-starved cells show enhanced expression of *MYC*, which is essential for proper utilization of glutamine. MYC inhibition prevented SG formation even with glutamine present, and increased cell death after arsenite exposure. We show that glutamine is crucial for cell viability under stress, and that its utilization for successful energy production requires the presence of G3BP1 and G3BP2 (collectively denoted G3BP1/2) proteins. Specially, we found that glutamine supports the generation of mitochondrial ATP, which is required for SG formation. However, neither glutamine nor glucose alone could supply the required energy levels, and both were needed for a successful stress response and for promoting cell survival. Altogether, we demonstrate not only that glutamine modulates SG formation under energy-limited conditions, but also that MYC and core SG proteins are involved in the shift to glutamine uptake during stress and contribute to cell survival.

## RESULTS

### Glutamine availability regulates SG formation in cancer cells

**Fig. 1.**
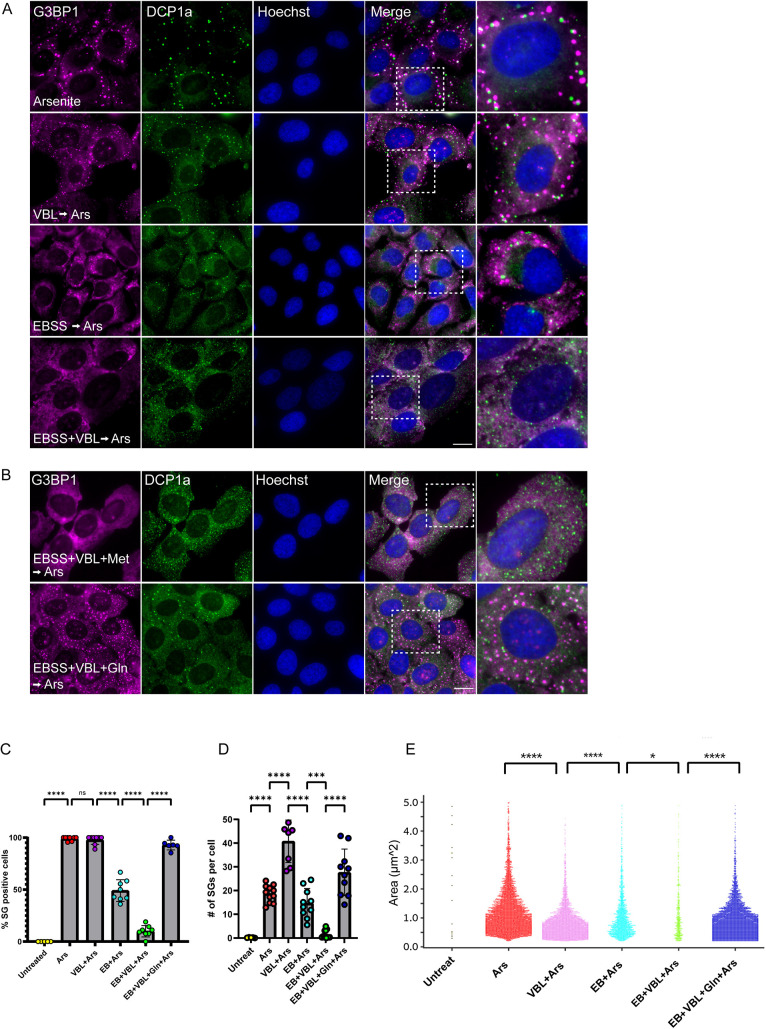
**Characterizing SG formation after metabolic and structural disruptions.** (A) Arsenite (Ars)-treated U2OS cells were stained by immunofluorescence with anti-G3BP1 as a SG marker (magenta) and anti-Dcp1a as a PB marker (green). Cells were supplemented with additional treatments of VBL to disrupt MTs or starved of AAs with EBSS medium. Together, these treatments prevented SG formation. Hoechst 33342 DNA stain is shown in blue. Dashed boxes indicating regions shown in the enlargements. (B) Cells treated with EBSS and VBL before arsenite, with either methionine (Met) or glutamine (Gln) added to the cell medium. Cells supplemented with glutamine showed a return of SGs to the cytoplasm, whereas the addition of other AAs had no effect. Scale bars: 20 µm. (C) Quantification of the population of SG-positive U2OS cells under the different treatment conditions. Data shown as mean±s.d., *n*=3 biological replicates with each circle indicating a field of cells. Data analyzed using one-way ANOVA with Tukey's post hoc test (ns, not significant, ****P<0.0001). (D) Counting of SGs in cells during the various treatments indicating number of SGs per cell. Data analyzed using one-way ANOVA with Tukey's post hoc test (***P<0.001, ****P<0.0001). Data shown as mean±s.d., *n*=3 biological replicates with each circle indicating a field of cells. (E) Swarm plots show individual SGs marked as a single point for each treatment. Each plot is distributed by size (area; µm^2^) of the SG. Glutamine supplementation showed not only a return of SG abundance, but specifically small SGs, given the presence of VBL in the culture. Number of granules were quantified for each treatment (*n*=3), 60–80 cells were analyzed for each group, with ∼6500 granules analyzed using two-tailed paired *t-*test (*P<0.05, ****P<0.0001).

Cancer cells can form SGs under a variety of stresses. It has been suggested that the formation of SGs could protect cancer cells from the stressors. We sought to determine stress conditions that would negate SG formation and thus reduce cell survival under stress. We focused on nutrient deprivation, a characteristic of the tumor environment, in conjunction with chemotherapy administration. Known stressors that can lead to SG formation in cancer cells are chemotherapies, such as the vinca alkaloid microtubule-disrupting agent Vinblastine (VBL) (Bartoli et al., 2011; Chernov et al., 2009; Fujimura et al., 2012; Ivanov et al., 2003; Nadezhdina et al., 2010; Schwed-Gross et al., 2022; Szaflarski et al., 2016; Wang et al., 2020). Destabilizing the microtubule network is expected to reduce cell survival of proliferating cancer cells, and VBL is employed in SG research given that the granules are known to move along microtubules which are essential for fusion of small SGs (Fujimura et al., 2009; Nadezhdina et al., 2010, Maucuer et. al., 2018). Sodium arsenite rapidly induces SG formation (0.5 mM) (Fig. 1A). VBL (300 nM) did not induce SG formation on its own (Fig. S1A). When given with arsenite, VBL did not halt SG formation, yet the granules formed were smaller and more dispersed throughout the cytoplasm compared to those formed in the presence of arsenite alone (Fig. 1A; Fig. S1A). We have previously shown that combining chemotherapy treatment with additional agents could alter SG assembly and/or disassembly and affect cell viability (Schwed-Gross et al., 2022). Specifically, vinca alkaloids given together with glucocorticoids were shown to enhance SG formation. Looking for a treatment that would instead limit SG formation when given together with vinca alkaloids, we chose to examine how interfering with cellular energy production pathways would affect SG formation.

Cancer cells require a high uptake of metabolites, including amino acids (AAs) (Butler et al., 2021). Because of this demand, nutrient deprivation is a key feature of the tumor microenvironment, which can yield metabolite-induced stress and even cell death (DeBerardinis and Chandel, 2016; Endicott et al., 2021; Green et al., 2014). We therefore examined how the lack of AAs would affect SG assembly under stress, especially when treated in tandem with the chemotherapy VBL. First, U2OS cells were starved for AAs by culturing in EBSS medium, which lacks AAs, for 18 h before exposure to arsenite. Serum was not added, because FBS supplementation is sufficient to prevent the cells from experiencing metabolic stress. AA starvation using EBSS medium impaired arsenite-induced SG formation, marked by a ∼50% reduction in SG-positive cells and an overall lower number of SGs per cell compared to that seen in unstarved cells (control) (Fig. 1A). Given that AA starvation can synergistically strengthen the efficacy of some chemotherapy treatments (Butler et al., 2021), the cells were simultaneously VBL-treated and AA-starved, leading to a further reduction in arsenite-induced SG formation, which ceased almost entirely (Fig. 1A). AA starvation or VBL treatment alone did not lead to SG formation (Fig. S1B). We also examined PBs under these conditions; these were detected, demonstrating that other cytoplasmic bodies were still capable of forming. Altogether, these findings suggest that AA starvation and VBL each moderately alter the ability of the cell to respond to stress and form SGs, but when combined, the metabolic and structural or cytoskeletal stressors significantly impair SG formation in a synergistic manner unseen with either treatment alone.

**Fig. 2.**
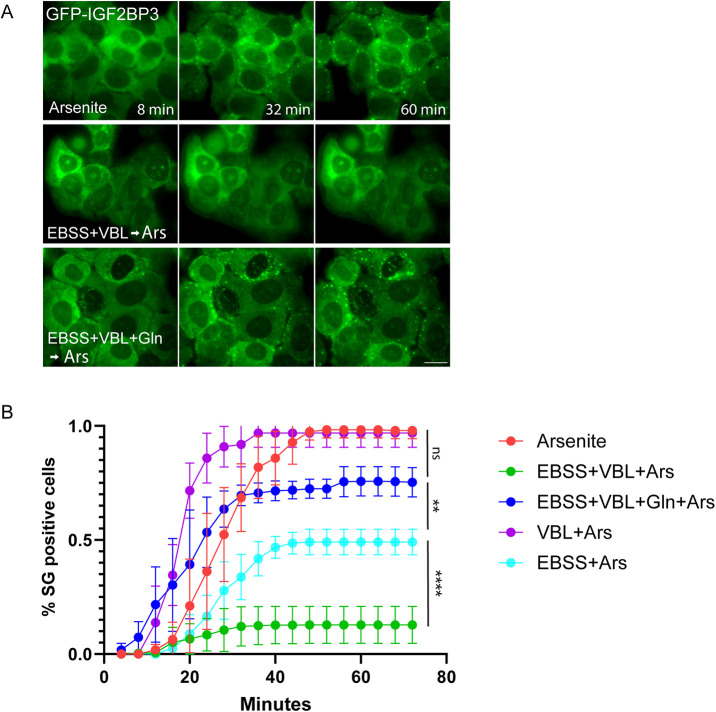
**Following the kinetics of SG formation in living cells.** (A) Quantification of SG formation in live-cell movies. Frames from movies of GFP–IGF2BP3-expressing cells imaged for 68 min every 4 min showing the formation of SG-positive cells over time during arsenite (Ars) treatment alone, during AA starvation+VBL, and during rescue by glutamine. Glutamine supplementation rescued SG formation in most cells. Scale bar: 20 µm. (B) Graph showing the percentage of SG-positive cells over the course of each treatment. Data shown as mean±s.d., *n*=4. Data were analyzed using two-tailed paired *t-*test (ns, not significant; **P<0.01; ****P<0.0001).

To identify the metabolites essential for SG formation, different AAs were reintroduced into the AA-depleted medium. Only the addition of glutamine completely restored SG formation, despite the lack of other essential AAs, and the microtubule network being dismantled (Fig. 1B; Fig. S2). Counting the SGs formed in fixed (Fig. 1C–E) and live cells (Fig. 2A,B), showed that under normal growth conditions, almost all cells were positive for SGs (Fig. 1C), with ∼20 SGs formed per cell during arsenite treatment (Fig. 1C,D). VBL treatment increased their numbers to ∼40 per cell, but their average size was reduced (∼1–2.5 µm versus <0.5 µm in diameter, respectively) (Fig. 1C–E). This indicates a lack of SG fusion due to the inability to anchor to and move along microtubules (Chernov et al., 2009; Nadezhdina et al., 2010). Cells that were AA-starved before arsenite addition were ∼50% positive for SGs in the cell population, with 10–15 SGs per cell. That number plummeted when cells were treated with both stressors, and AA-starved+VBL-treated cells were only ∼10% positive for SGs. Cells that did succeed in forming SGs, made very few (∼5). Reintroducing glutamine, significantly increased the number of SGs and the percentage of SG-positive cells, but did not affect SG size (Fig. 1C–E). The finding that the glutamine-restored plot in Fig. 1E (SG size) resembled that of the VBL-treated plot, demonstrates that glutamine can restore the energy-related characteristics of SGs, rather than the structural ones. Cells that received the different treatments, but without arsenite, did not form SGs (Fig. S1B). Imaging of SG formation in living cells expressing GFP–IGF2BP3 confirmed SG formation in most of the cells exposed to arsenite under control conditions, whereas AA-starved+VBL-treated cells formed very few granules (Fig. 2). In contrast, AA-starved cells formed SGs in ∼50% of the cells, and in VBL-only treated cells, 100% of the cell population formed SGs. However, ∼75% of the cells that were replenished with glutamine showed SG formation upon arsenite addition (Fig. 2A,B; Movies 1–3; a summary of treatments and their effect on SG formation can be found in Table S1). Altogether, this implies that glutamine influences SG formation through a metabolic pathway that is independent of cytoskeletal integrity, and that glutamine is capable of single handedly restoring the SG phenotype in nutrient-depleted cells.

**Fig. 3.**
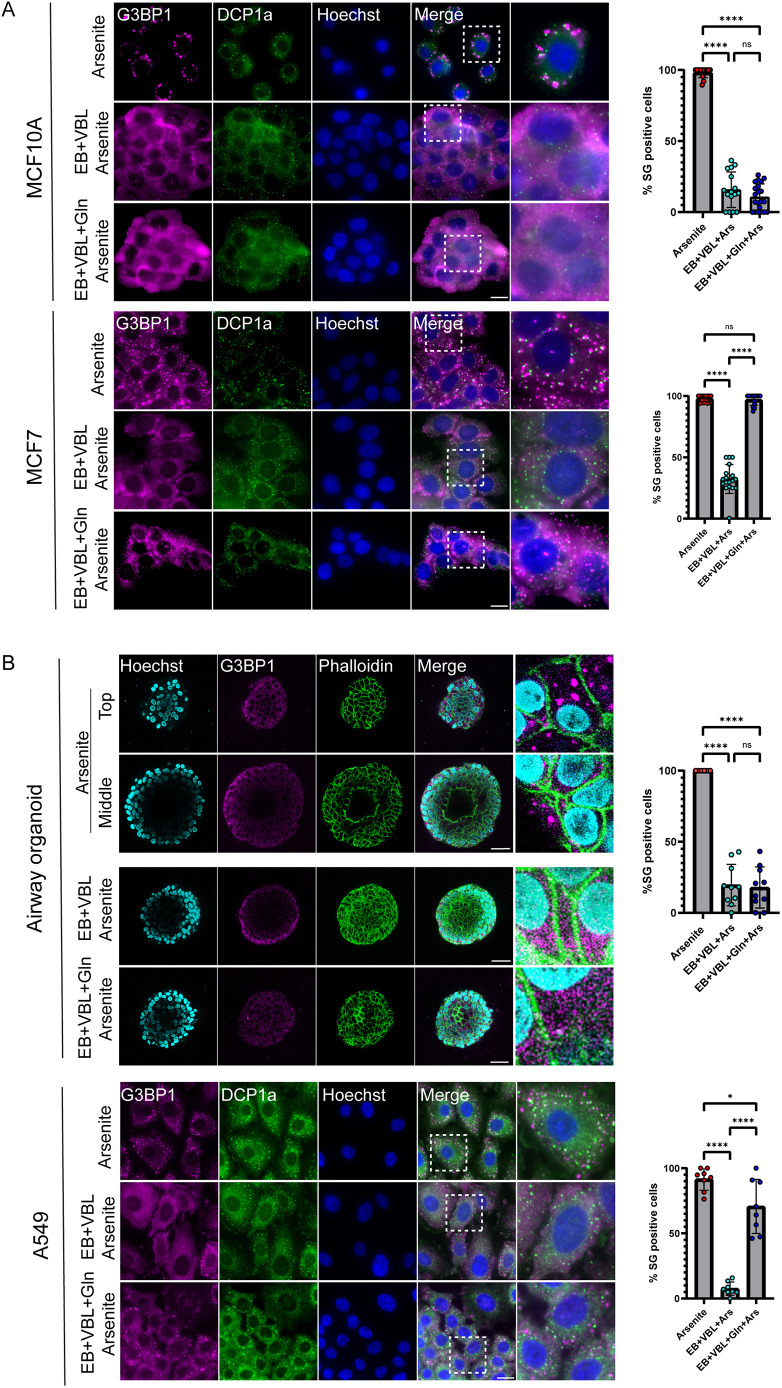
**Cancer cells show reliance on glutamine for SG formation under starvation conditions.** (A) MCF7 and MCF10A cells treated with arsenite or EBSS (EB)+VBL followed by arsenite, with or without glutamine, then stained by immunofluorescence with anti-G3BP1 as a SG marker (magenta) and anti-Dcp1a as a PB marker (green). Only the cancerous MCF7 cells showed SG formation after glutamine supplementation. Hoechst 33342 DNA stain is shown in blue. Dashed boxes indicating regions shown in the enlargements. Scale bars: 20 µm. (B) Human primary-derived airway organoids treated with arsenite or EBSS+VBL followed by arsenite, with or without glutamine, were stained with anti-G3BP1 as a SG marker (magenta). Phalloidin staining of actin (green) was used to detect the cell outline. Top panels show an airway organoid treated with arsenite, showing the top and middle of the organoid with a lumen visible inside the organoid sphere. Scale bars: 36 µm. Bottom panel shows the starved groups, where no difference in the number of stress granules could be seen between those with and without the addition of glutamine. Scale bar: 40 µm. A549 lung adenocarcinoma cells were subjected to the same treatments as above. Cancer cells showed recovery of the SG population after addition of glutamine. Scale bar: 20 µm. For the presented quantifications, data shown as mean±s.d., *n*=3 of the cell population that was positive for SGs. Data were analyzed using one-way ANOVA with Tukey's post hoc test (ns, not significant; *P<0.05; ****P<0.0001).

Given that many types of cancer cells are addicted to glutamine, we assessed whether the recovery of SG formation upon glutamine supplementation was unique to cancer cell biology (Fig. 3). This was achieved by subjecting cancerous human breast epithelial cells (MCF7) and similar non-cancerous breast epithelial cells (MCF10A) to the above treatments and to glutamine recovery. We identified SG formation in both cell lines after exposure to arsenite. Conversely, both cell lines showed significantly reduced numbers of SGs after an AA-starvation and VBL combination treatment. However, the cancerous MCF7 cells did recover SG formation after glutamine was reintroduced to the cell culture, whereas the MCF10A cells did not (Fig. 3A). Similar results were obtained for other human and murine cancerous and non-cancerous lines (Fig. S3). The effect of glutamine was also examined in human-derived airway organoids generated from non-cancerous lung tissue, and in A549 human lung carcinoma cells (Fig. 3B). Taken together, our findings show that cancer cells uniquely utilize glutamine for the formation of SGs under starvation and oxidative stress.

### Glutamine recovery prompts global gene rewiring to contend with cellular stress

**Fig. 4.**
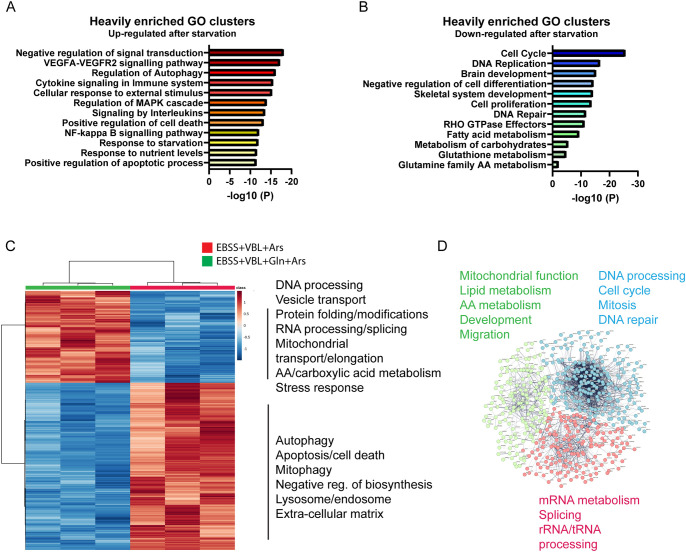
**Gene enrichment analysis after glutamine rewiring.** (A,B) Gene Ontology (GO) networks upregulated (red scale) and downregulated (blue scale) after AA starvation+VBL and arsenite, compared to arsenite-treated U2OS cells. Upregulated genes indicate the cells are in a highly stressed state, and repressed genes are related to growth, development and metabolism. (C) Heatmap indicating the top 1000 most heavily changed genes in AA-starvation+VBL treated cells compared to those supplemented with glutamine. Many of the processes restarted after glutamine supplementation are the same pathways downregulated after starvation. (D) STRING network cluster analysis showing genes upregulated after glutamine recovery. Genes cluster into three main groups; DNA and cell cycle, RNA processing and metabolism and development. Results obtained for *n*=3 libraries prepared for each treatment group.

Given that SG formation is one of the many ways cells contend with stress, we applied RNA sequencing to investigate the global changes in the stress response to AA starvation and glutamine rescue. Gene ontology enrichment analysis of the NGS data was performed on genes that were either significantly upregulated or downregulated in arsenite-treated cells (control) compared to the AA-starvation+VBL-treated cells prior to the arsenite treatment (Fig. 4A,B; Table S2). Many genes associated with autophagy, apoptosis and cell death were highly enriched in the AA-starved cells. Many immune and cytokine pathways were also upregulated. In contrast, genes related to the cell cycle, growth and metabolism were severely repressed, confirming that the cells were in an acute state of stress and metabolic depletion.

An examination of the change in the gene expression profile before and after the glutamine recovery revealed large clusters of both upregulated and downregulated genes (Fig. 4C; Table S3). The heatmap shows the top 1000 altered genes. The reintroduction of glutamine led to the activation of pathways related to metabolism, cell cycle, mitosis, RNA processing and DNA synthesis. Downregulated pathways were autophagy, cell death and biosynthesis. Glutamine partially reversed the pathways upregulated or downregulated by AA starvation. Thus, besides promoting an increase in available energy, the addition of glutamine alone can dictate a whole variety of cellular processes linked to the stress response. This analysis demonstrates the larger cellular context in which SGs operate and highlights other elements that contribute to cell survival after glutamine recovery.

### Glutamine and glucose are both essential for SG formation

A K-means cluster analysis (STRING network analysis) showed dense clusters of upregulated genes after glutamine addition (Fig. 4D; Table S4). One network consisted of genes related to metabolism, AA synthesis, carboxylic acids and the TCA cycle, as well as migration and various components of cellular development (Fig. S4A). Finding that glutamine was required for SG formation uniquely in cancer cells and establishing that many metabolic pathways were altered by glutamine supplementation, led us to investigate specific changes in metabolic responses and energy generation driven by glutamine, and the effect on SG formation in cancer cells.

**Fig. 5.**
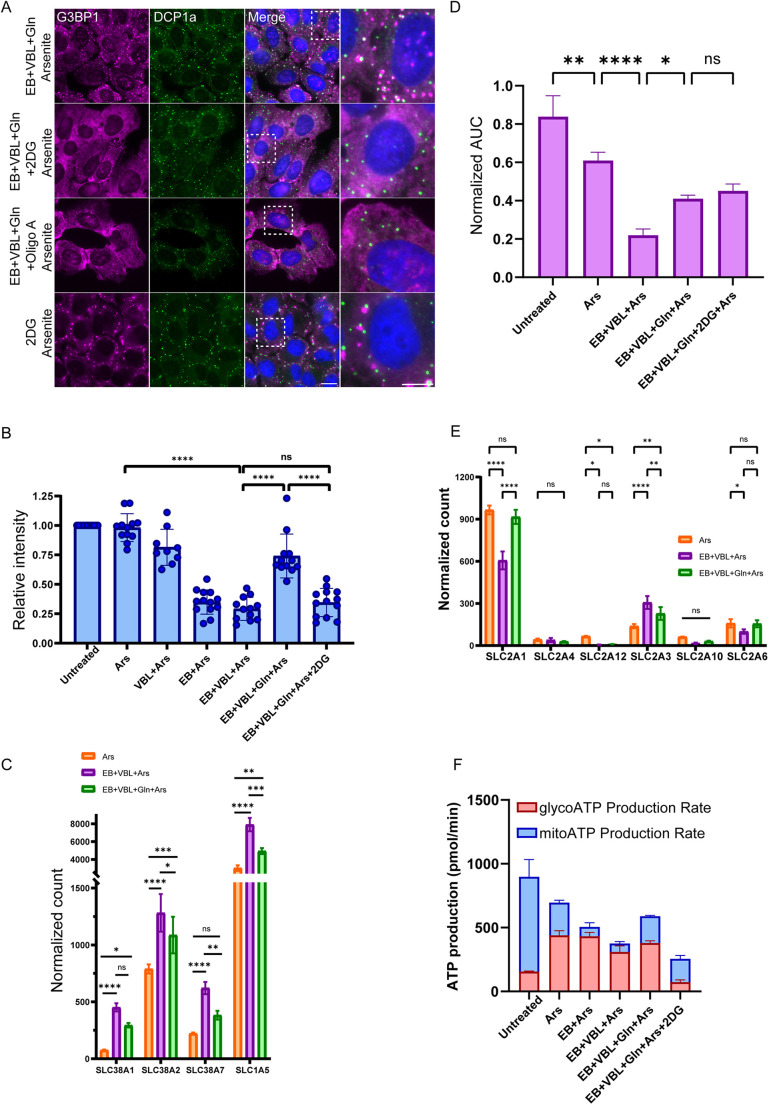
**Metabolic profile of cells during starvation and glutamine recovery show requirement of dual input of glutamine and glucose.** (A) U2OS cells were AA-starved (EBSS, EB)+VBL, along with glutamine and then arsenite, followed by immunofluorescence staining with anti-G3BP1 as a SG marker (magenta) and anti-Dcp1a as a PB marker (green). Additional ATP inhibitors were added to the cell culture. 2-deoxy-glucose (2DG) completely inhibited SG formation in glutamine recovered cells, the addition of oligomycin A had the same effect. Hoechst 33342 DNA stain is shown in blue. Images representative of three repeats. Scale bar: 20 µm. (B) ATP measurements under the different treatment conditions show significant increase in ATP levels after glutamine supplementation in starved cells (*n*=10). (C) NGS profile of glutamine uptake transporters show an increase of the transporters in AA-starved and glutamine-recovered conditions. (D) NBDG glucose uptake assay showed a decrease in glucose uptake following arsenite exposure, a steep decline after AA-starvation (EB)+VBL treatment, but an increase after glutamine supplementation (*n*=3). AUC measures 9.3 min retention time signal, normalized to cell number. (E) NGS profile of glucose uptake transporters. Transporters decreased in starved conditions and rose again with the addition of glutamine. All analyses in B–E presented as mean±s.d., *n*=3, except in B where *n*=10 with statistical analysis undertaken using nested one way-ANOVA with Tukey's post hoc test (*P<0.05, **P<0.01, ***P<0.001, ****P<0.0001, ns, not significant). (F) Seahorse XF ATP rate assay for quantifying ATP levels originating from glycolytic (red) and mitochondrial (blue) fractions (mean±s.d., *n*=3). Glutamine increased overall ATP levels, but exclusively from the mitochondrial fraction.

First, other stressors were tested under the starvation and glutamine recovery conditions to confirm that the effects observed were not exclusively linked to potential metabolic effects of arsenite. ER stress (thapsigargin) and another oxidative stress (hydrogen peroxide; H_2_O_2_) led to no SG formation for AA-starved+VBL-treated cells. Under these conditions the cells succeeded in forming SGs after the addition of glutamine (Fig. S5A,B). Next, we examined the pathways that generate ATP. Cellular energy demands are mainly supplied by ATP generation through glycolysis or mitochondrial respiration. Notably, glutamine is a major energy source in cancer cells and an important supplier of the TCA cycle (Ardawi and Newsholme, 1983; Newsholme et al., 1985; Reitzer et al., 1979). As cancer cells are also highly dependent on glucose (DeBerardinis and Cheng, 2010; Jin et al., 2023), in addition to the influence of glutamine, we also tested the effect of glucose-generated ATP on SG formation, by treating cells 20 min before arsenite addition with 2-deoxy-glucose (2DG, 20 mM) a glucose analog that cannot be utilized by the cell and limits glycolysis-generated ATP. In AA-starved and VBL-treated cancer cells that were then treated with glutamine and 2DG, SGs did not form (Fig. 5A). In addition, when ATP synthase was inhibited with oligomycin A, the addition of glutamine did not result in SG formation due to lack of ATP synthesis (Fig. 5A). 2DG alone did not noticeably impair SG formation, suggesting that limiting glycolysis does not affect SG formation on its own (Fig. 5A). Altogether, we find that when rescuing SG formation with glutamine under AA-starvation conditions, glycolysis inhibition halts SG formation, despite glutamine being present. These results show that glucose and glutamine metabolism are intertwined, and both pathways are essential for SG formation under energy-limited conditions.

### Uptake of glutamine affects glucose uptake for energy production

Before identifying the specific effects of these metabolites on SG formation, we first needed to discern the general effects of glutamine and glucose on energy levels in the cancer cells using ATP quantification assays. Untreated and arsenite-treated cells showed similar ATP levels, indicating that arsenite alone does not significantly lower energy levels (Fig. 5B). Cells treated with both VBL and arsenite showed a moderate decrease in ATP levels. The decline was far more pronounced in cells exposed to AA starvation and arsenite. The reintroduction of glutamine to these cells increased their ATP levels significantly. When glucose metabolism was subsequently impaired in these cells by the addition of 2DG, ATP levels again declined even though glutamine was added (Fig. 5B). Control conditions with the treatments alone but without arsenite are shown in Fig. S4B. Of note, the ATP levels in the treated cells during glutamine recovery (Fig. 5B) were double those of cells with glucose but no glutamine (i.e. in AA-starved cells) and those of cells with glutamine but no glucose (i.e. glutamine rescued and 2DG treated). This means that neither glutamine nor glucose alone can fully supply the energy demands of stressed cells and suggests that an energy threshold must be crossed to allow SG formation.

Given that cells under stress would need to enhance glutamine uptake to supply ATP requirements, we examined the expression levels of all major glutamine uptake transporters using the transcriptomics information obtained above from the AA-starved and glutamine-recovered cells, and arsenite-treated cells (Fig. 5C). Higher expression levels were detected and, notably, the premier glutamine transporter SCL1A5 had almost three-fold higher expression than other transporters. This increase was also observed on the protein level (Fig. S6A). This implies that when energy levels fall due to the AA starvation stress, cancer cells respond by increasing the expression of glutamine transporters as a means to maximize glutamine intake in order to meet its metabolic demands.

The finding that inhibiting glycolysis alone does not affect SG formation, but that this inhibition does negate the rescue by glutamine (Fig. 5A,B), suggests that glucose metabolism towards ATP production must also be involved in SG formation, and that glucose uptake might be a limiting factor. To quantify glucose uptake levels in stressed cells, a glucose uptake assay that relies on fluorescently labeled glucose (NBDG) was applied to the various treated and untreated cells. Untreated cells exhibited high glucose uptake levels, which declined upon exposure to arsenite (Fig. 5D), as expected (Castriota et al., 2020). Interestingly, AA-starved+VBL-treated cells showed very low glucose uptake, whereas the glutamine-recovered cells showed significantly elevated glucose uptake, although lower than that of the arsenite-treated cells. This indicates that glutamine not only promotes its own uptake but also increases the uptake of glucose.

Previous studies have shown a metabolic rewiring to glutamine under starvation conditions (Le et al., 2012; Wise et al., 2008; Yuneva et al., 2007). The sequencing data obtained showed a significant decrease in the major glucose uptake transporter SLC2A1 under AA starvation conditions (Fig. 5E), which resulted in lower levels of glucose uptake and ATP production. Other less significant transporters had relatively low levels of expression. Altogether, the data suggest that very low levels of metabolites, and subsequently low energy levels, drive the cell to invest significant assets in glutamine uptake, even at the expense of glucose uptake.

### ATP output from glycolysis and mitochondrial respiration is required for SG formation

Given that lack of glutamine limited SG formation (Fig. 1) and that parts of the SG assembly pathway are dependent on ATP (Eum et al., 2020; Jain et al., 2016), we hypothesized that for SG formation, an energy threshold must be passed, and that both glutamine and glucose are essential for reaching this threshold. The energy supplied through glucose uptake could originate from either glycolysis or mitochondrial respiration. To test our hypothesis, we had to distinguish between the ATP coming from these two energy sources, and therefore utilized the Seahorse XP ATP rate assay. These measurements demonstrated that the highest energy output was observed in untreated cells. Because these cells had fully functional mitochondria, a larger proportion of the ATP output came from mitochondrial production (Fig. 5F). Given that arsenite damages mitochondria (Ding et al., 2023; Prakash et al., 2016; Zhong et al., 2022), this reduced the amount of ATP originating from them, and AA starvation reduced mitochondrial ATP production even more owing to the lack of metabolites.

In line with the known support glutamine plays in the TCA cycle, we detected a significant increase in mitochondrial ATP production after the reintroduction of glutamine to the starved cells (Fig. 5F). Lending further support to the previous ATP assay findings (Fig. 5B), the glutamine-recovered cells contained ATP from both glucose and glutamine inputs. This was more starkly observed in the AA-starved cells (containing glucose), in which almost all the ATP was generated by glycolysis, whereas the ATP fraction that oscillated between the various treatments came from the mitochondria (Fig. 5F). However, when glycolysis was inhibited by 2DG, an additional drop in ATP levels was observed, which was exclusive to the glycolytic component. Notably, because glutamine was present, the mitochondrial fraction did not change. This analysis confirmed that not only is SG formation an energy-dependent process, but that there indeed is a threshold of available energy required for the cell to form SGs under starvation conditions. When the cellular energetic supplies are stripped down to the bare minimum, neither of the metabolites is sufficient on their own, and ATP stemming from both glycolytic and mitochondrial fractions is required to cross the energy threshold to successfully form SGs.

### *MYC* expression is required to shift cells to glutamine uptake

**Fig. 6.**
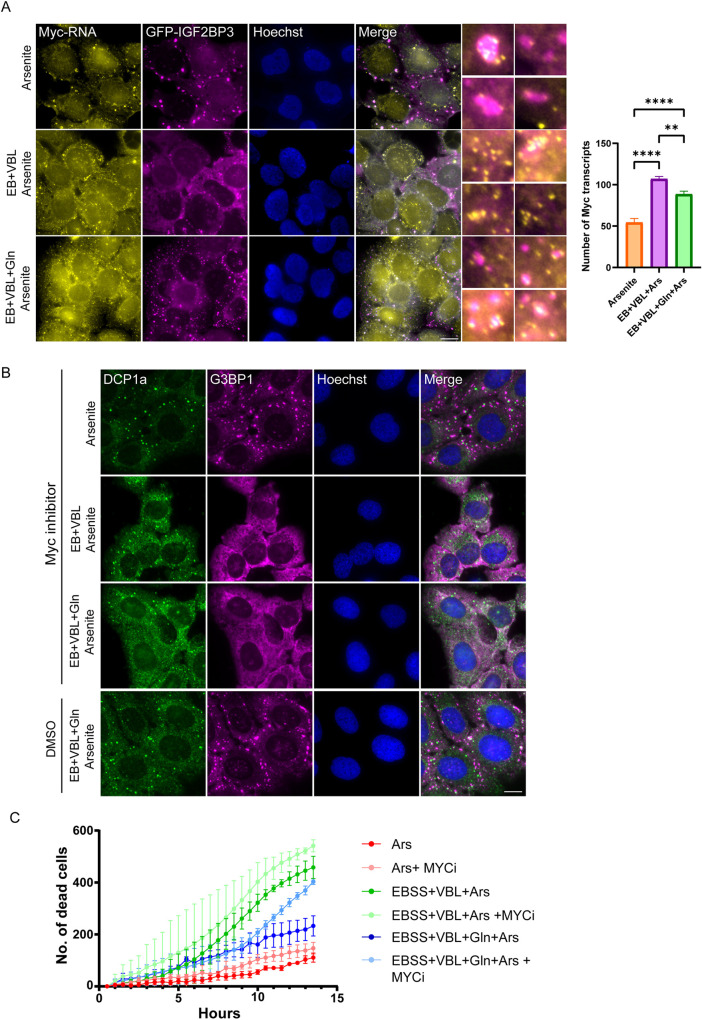
**MYC regulates metabolic rewiring during glutamine recovery.** (A) RNA FISH in GFP–G3BP1 expressing (magenta) U2OS cells to detect endogenous *MYC* transcripts (yellow), showing *MYC* transcripts in SGs, and increased *MYC* levels after treatments (VBL; EB – EBSS medium). Hoechst 33342 DNA stain is shown in blue. Scale bar: 20 µm. (Right) Endogenous *MYC* transcripts were quantified (mean±s.e.m., *n*=3). AA-starved (EB)+VBL treated cells show the highest levels of *MYC* transcripts, with significant reduction after glutamine addition. Data were analyzed using one-way ANOVA followed by Tukey's post hoc test (**P<0.01, ****P<0.0001). (B) U2OS cells were AA-starved (EB)+VBL, along with glutamine addition and then arsenite and a MYC inhibitor (MYCi), followed by immunofluorescence staining with anti-G3BP1 as a SG marker (magenta) and anti-Dcp1a as a PB marker (green). The presence of the MYC inhibitor prevented all SG formation in AA-starved cells even with glutamine present. Control with DMSO shows cells can form SGs readily without the inhibitor present. Hoechst 33342 DNA stain is shown in blue. Images representative of three repeats. Scale bar: 20 µm. (C) Quantifications of dead cells over time measured by the Cytotox NIR dye under various treatments and arsenite exposure (0.625 µM, sufficient to form SGs), both in U2OS cells with (light curves) or without (dark curves) a MYC inhibitor. Presence of the MYC inhibitor dramatically increased cell death in AA-starved cells, even with glutamine present (mean±s.d., *n*=3 biological replicates).

MYC is a known regulator of glutamine metabolism (Csibi et al., 2014; DeBerardinis et al., 2007; Wise et al., 2008; Yuneva et al., 2007). It has been reported to be a major TCA stabilizer, achieved by causing the conversion of glutamine to glucose (Wise et al., 2008). The transcriptomics data revealed that *MYC* was one of the most upregulated genes in AA-starved+VBL-treated cells that had shifted to glutamine uptake (Fig. S4C). *MYC* transcript levels in single cells were then measured by single-molecule RNA FISH, which demonstrated higher *MYC* mRNA levels in AA-starved cells compared to that in untreated cells (Fig. 6A). *MYC* transcripts were associated with SGs in arsenite-treated cells, which often contained one or two *MYC* mRNAs. In AA-starved+VBL-treated cells, in which SGs failed to form, *MYC* mRNA was highly abundant throughout the cytoplasm. Conversely, in the glutamine-recovered cells, many of these transcripts were associated with SGs (Fig. 6A). The glutamine-recovered cells contained lower levels of *MYC* mRNA compared to the AA-starved ones, and their glutamine and glucose uptake transporters correspondingly increased or decreased (Fig. 5C,E). To further verify the changes in MYC levels, a luciferase assay was performed with a reporter plasmid that contains 4 E-box sequences, the canonical MYC-binding sites, to measure the levels of MYC binding and transcriptional activation under the various conditions. Increased levels of MYC activity were found under the conditions that had more MYC expression (Fig. S4D). MYC protein levels showed a similar pattern to gene expression levels, but with less drastic changes between groups, suggesting that mild changes at the protein level can alter MYC activity (Fig. S6B). Altogether, *MYC* expression proved to be a reliable indicator of glutamine or glucose consumption, and the results suggest overexpressed *MYC* in starvation conditions is able to drive glutamine uptake and utilization, as a means to contend with the high energy demands of the cell.

To examine the importance of MYC in this metabolic transition, the commercial MYC inhibitor 10058-F4 was used to inspect its effects on SG formation during the various conditions. As expected, MYC inhibition did not dramatically hinder SG formation in arsenite-treated cells in full growth medium (Fig. 6B), probably because several alternative forms of energy are readily available. SG formation was entirely inhibited by the MYC inhibitor in AA-starved and VBL-treated cells. Although under these conditions mature SGs failed to fully form in most cells even without the MYC inhibitor, this inhibition prevented even the smallest nucleation events of SG formation (Fig. 1A). Most surprising was the result of MYC inhibition in cells that received glutamine. Although these cells can form SGs in high numbers (Fig. 1B), after MYC inhibition no SGs formed (Fig. 6B), demonstrating that MYC is indeed directly responsible for utilization of glutamine to form SGs.

To understand the functional relevance of halting SG formation after MYC inhibition, a cell death assay was performed to examine cell viability over the course of treatment (Fig. 6C). Arsenite-treated cells began dying ∼6 h post treatment, and the number of dead cells was slightly elevated after MYC inhibition (red curves, Fig. 6C). AA starved+VBL-treated cells began dying at ∼5 h, and number of dead cells rose much more rapidly, but showed almost no change after MYC inhibition (green curves, Fig. 6C), for no glutamine was present. But when glutamine was added, the rate of cell death was reduced (blue curve, Fig. 6C). This increase in cell survival after glutamine conforms with the various reductions in inflammation and apoptosis pathways seen in the next-generation sequencing (NGS) analysis, as well as the increase in metabolic and other anabolic activities (Fig. 4). However, in this case MYC inhibition had an effect, and cell death rates were much faster (compare blue colored curves in Fig. 6C). This indicates that the lack of MYC activity severely limits the effective protection conferred by the glutamine. The plot demonstrates two clusters, in the bottom three treatments, the cells are capable of forming SGs with slow linear progression of cell death, whereas in the top three treatments the cells are unable of forming SGs, and rapid rates of death are observed. Altogether, the data show that: (1) MYC is essential for cell survival after arsenite exposure in cells that rely on glutamine; (2) this metabolite in turn is needed for ATP production that will mount a successful stress response; and (3) that cell survival correlates to the presence or absence of SGs.

To advance the gene expression analysis, gene set enrichment analysis (GSEA) was implemented and called to attention two related but divergent MYC activity pathways (Fig. S4E). Both the starved cells, and those with the glutamine supplementation showed high levels of MYC, and the GSEA analysis highlighted that the cells with glutamine were enriched for ‘MYC targets V1’, which modulate stress response, cell cycle, and RNA processing. However, the AA-starved+VBL-treated cells were enriched in ‘MYC targets V2’, which primarily increase translation machinery, including ribosomal subunits and rRNA processing, likely acting as a mechanism to address the severe lack of protein available after starvation. We conclude that whereas MYC is a crucial protein in the stress response, the targets of MYC are actually pliable given the metabolic situation.

### G3BP1/2 proteins are required for glutamine utilization and cell survival

**Fig. 7.**
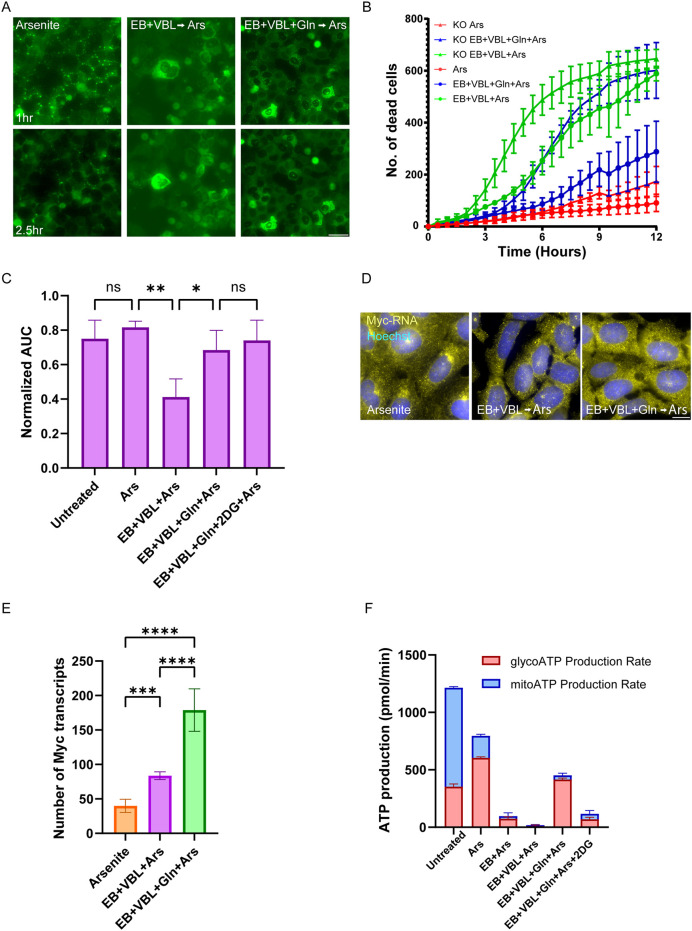
**Glutamine recovery and metabolic rewiring occur in a G3BP1/2-dependent manner.** (A) Fluorescence images captured in live U2OS cells stably expressing GFP–IGF2BP3. Treatments capable of forming SGs lead to granules formation after ∼1 h, and granule dissolving after ∼2.5 h. Images representative of three repeats. Scale bar: 20 µm. (B) Quantifications of dead cells over time measured by the Cytotox NIR dye under various treatments and arsenite exposure (0.625 µM), both in WT U2OS cells (circles) and ΔΔG3BP1/2 (triangles). WT cells that received glutamine supplementation showed significant increase in cell survival, whereas ΔΔG3BP1/2 cells showed increased cell death in every treatment category (mean±s.d., *n*=3). (C) NBDG glucose uptake assay in ΔΔG3BP1/2 cells. ΔΔG3BP1/2 cells showed increased glucose uptake compared to WT cells displayed in Fig. 4. Data were analyzed using one-way ANOVA with Tukey's post hoc test (*P<0.05; **P<0.01; ns, not significant). (D) smRNA FISH in ΔΔG3BP1/2 U2OS cells to detect endogenous *MYC* transcripts (yellow), overlayed with Hoechst 33342 DNA stain (blue). Scale bar: 20 µm. (E) Quantification of *MYC* transcripts using smRNA FISH in ΔΔG3BP1/2 cells (mean±s.d., *n*=3). KO cells showed decreased *MYC* expression in arsenite and starved groups compared to WT cells (see Fig. 6A)). (F) Seahorse XF ATP rate assay for quantifying ATP and dividing into glycolytic (red) and mitochondrial (blue) fractions in ΔΔG3BP1/2 cells. KO cells showed little ATP coming from the glycolytic fraction, and even glutamine supplementation, which increases overall ATP, did not change mitochondrial ATP production. Data are mean±s.d., *n*=3.

To address further the connection between the prevalence of SGs and cell survival during long-term exposure to oxidative stress, we examined SG formation in living cells over time. Time-lapse movies of cells exposed to arsenite after treatment showed that SGs formed after ∼1 h from arsenite administration and then dissolved within the next 3 h (Fig. 7A). We hypothesized that this time window of SG formation is important for cell survival, and that the ability of the cell to contend with the stresses due to the presence of glutamine is achieved via SG core G3BP proteins. Therefore, double knockout (KO) of G3BP1 and G3BP2 (ΔΔG3BP1/2) cells that cannot form SGs upon arsenite treatment were used (Kedersha et al., 2016). Living cells were imaged over long periods and the number of dead cells was quantified over time under the different treatments (Fig. 7B). Moderate cell death was seen after ∼9 h of arsenite treatment (red curves, Fig. 7B), whereas AA-starved+VBL-treated cells without glutamine began dying much earlier, at ∼4 h at much higher rates (green curves, Fig. 7B). The addition of glutamine reduced cell death in the wild-type (WT) cells (WT blue curve, Fig. 7B). However, there was a stark difference between the WT and KO cells. AA-starved KO cells began dying after less than 3 h, and the addition of glutamine to these cells only mildly extended their lifespan and did not alter the rate of death (KO blue curve, Fig. 7B). The ΔΔG3BP1/2 cells with glutamine supplementation began to die at ∼4 h, and with a remarkably similar trajectory to WT AA-starved cells that did not contain glutamine (compare WT green curve to KO blue curve, Fig. 7B). To confirm that the lack of G3BP1/2 was responsible for these effects on cell viability, rescue experiments were performed using knockout cells that expressed GFP–G3BP1 (Marmor-Kollet et al., 2020). These cells showed significantly less cell death with the glutamine supplement, and displayed curves that were similar to the WT cells (Fig. S7A). This suggests that the glutamine pathway is involved in SG formation, and that cell survival through glutamine requires the presence of the G3BP1/2 proteins.

Given that the recovery by glutamine was found to act through G3BP1/2, we suspected that a lack of G3BP would lead cells to try compensating for the lack of glutamine through the glucose pathway. To this end, glucose uptake was examined in the KO cells. Untreated KO cells showed identical glucose uptake to WT U2OS cells [a value of ∼0.8 for the area under curve (AUC)] (Fig. 7C). However, there was no reduction in glucose uptake in the KO cells treated with arsenite (0.8 AUC), in contrast to what was observed with the WT cells (0.6 AUC) (Fig. 5D). This indicates that although arsenite changes glucose homeostasis, it does so in a manner related to SG formation, and in a G3BP1-dependent manner. Similarly, there was significantly elevated glucose uptake in KO cells under AA-starved conditions that received glutamine supplementation (0.4 AUC) compared to the much lower glucose uptake in the WT cells (0.2 AUC). However, although glucose uptake was elevated in AA-starved+VBL-treated KO cells compared to WT cells, the KO cells still displayed significantly reduced glucose uptake compared to that seen other treatment groups (Fig. 7C), probably due to reduced cell viability. The KO cells that received the glutamine supplement also showed increased glucose uptake (0.7 AUC) compared to WT cells (0.4 AUC). Thus, in every condition, the KO cells show increased glucose uptake compared to WT cells when exposed to arsenite, together with decreased cell viability conferred from glutamine supplementation. KO cells rescued with GFP–G3BP1 displayed the same significant differences that the WT cells showed during the treatments, but did display somewhat overall elevated glucose uptake (Fig. S7B). We conclude that KO of core SG proteins shifts cells to rely more heavily on glucose, and thus they do not utilize the glutamine present in the culture to contend with oxidative stress.

### G3BP1/2 proteins are linked to *MYC* expression controlling ATP production pathways

Because we have established a link between changes in glucose uptake and levels of *MYC* expression, we also expected a concomitant change in the levels of *MYC* transcripts in the KO cells. ΔΔG3BP1/2 cells under arsenite and AA-starved conditions had increased glucose uptake compared to the WT cells and showed a decrease in the number of *MYC* mRNAs from ∼110 to ∼80 individually detected mRNAs (Fig. 7D,E). In the rescued cells, MYC expression levels returned to their original pattern (Fig. S7C). The KO cells also showed lower levels of MYC activity in the luciferase assay (Fig. S4D), while also not showing any significant increase in the level of MYC protein after AA starvation (Fig. S6B). This implies that WT cells could better utilize glutamine to support ATP production, whereas KO cells relied more heavily on glucose uptake, and therefore expressed lower levels of *MYC*. Thus, the KO of G3BP1/2 seems to promote cell death under AA-starved conditions not only due to the absence of the granule but also due to the change in the metabolic profile of the cell, from reliance on glutamine to dependence on glucose.

Such a dramatic change in glutamine and glucose metabolism should be reflected in the energy levels obtained from mitochondria or glycolysis, respectively. To explore this issue, we employed the Seahorse XF ATP rate assay to differentiate between the mitochondrial and glycolytic ATP fractions in the KO cells. As seen in the WT cells, untreated KO cells had the highest energy output, resulting from their unstressed mitochondria. This demonstrates that under homeostatic conditions, WT and KO cells behave in an identical manner. These cells showed elevated glycoATP, which was unsurprising given the enhanced glucose uptake observed across the different treatments. Upon arsenite induction, ΔΔG3BP1/2 cells also produced more ATP from glycolysis, corresponding to the enhanced glucose uptake (Fig. 7F). AA-starved+VBL-treated KO cells showed severely diminished ATP output, correlating with the increased cell death observed (Fig. 7B).

Surprisingly, when glutamine was reintroduced, the ATP levels increased, a rise that came exclusively from the glycolytic fraction (Fig. 7F). This implies that when G3BPs are missing, glutamine supports certain cellular pathways but is not utilized to support the TCA cycle (in contrast to what is seen in WT cells), for there is an increase in the overall ATP but not in the mitochondria-generated ATP fraction. This failure to produce mitochondrial ATP comes at the expense of a satisfactory metabolic output. The KO cells treated with 2DG had no glycolytic fraction, leaving the cells with almost no ATP production and no SGs, resulting in high rates of cell death. The G3BP1-rescued cells showed identical patterns to WT cells, and displayed an expected rise in mitochondrial ATP after the addition of glutamine, not seen in the knockout cells (Fig. S7D). Altogether, we find that the ability of a cell to survive stress is driven by continuous ATP generation by both the glutamine and glucose metabolic pathways. In WT cells, stress causes SG formation through G3BPs, which, in turn, are required for cell survival. KO of the G3BPs results in an inability to form SGs, as well as a shift away from the normal metabolic response to starvation, leading to diminished survival and ultimately unsatisfactory metabolic output, culminating in a failure to utilize glutamine to support the mitochondrial ATP production.

## DISCUSSION

**Fig. 8.**
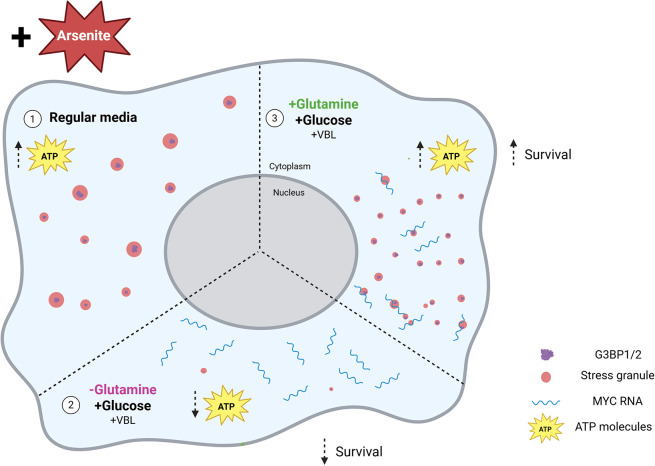
**Illustration of the suggested model.** On left (marked 1), cells form large SGs after exposure to arsenite, with adequate amounts of ATP present. On bottom (marked 2), cells lack sufficient ATP and thus fail to form SGs and display decreased survival after arsenite exposure. On right (marked 3), after adding glutamine to media, cells cross the energy threshold with glucose and glutamine present, yielding increases in ATP necessary to form SGs. This results in greater cell survival. Mechanistically this is linked to increase in the expression of *MYC*, which regulates the transition to glutamine metabolism, and this process is mediated by core SG proteins G3BP1/2.

Although studies have determined that glutamine is a key metabolite in cancer cells (DeBerardinis and Cheng, 2010; Medina et al., 1992; Reitzer et al., 1979), its direct connection to SG formation, and how this, in turn, promotes cell survival have not yet been fully characterized. In this study, we examined the role of glutamine in the cancer cell stress response, focusing on the intersection of stress granule formation and cell metabolism. Under conditions that limit SG formation, glutamine can single-handedly restore the SG population (Fig. 8). As attested by previous work on SGs and the cytoskeleton (Chernov et al., 2009), dismantling the microtubule network (by VBL) starkly changed SG morphology and decreased SG size, thereby increasing the number of granules in the cell. However, in the case of limiting the amino acid pool (by growing cells in AA-depleted media; EBSS), the result is lower rates of SG formation and fewer SG-positive cells overall. The simultaneous use of both stresses – structural/cytoskeletal from the chemotherapy and energy-disrupting agents mimicking nutrient deprivation of the tumor, prevented granule formation. ATP assays confirmed that the absence of granules was mechanistically linked to a lack of available energy resources. Surprising was the outsized effect glutamine had on energy levels, and in turn on granule formation. The addition of this metabolite alone could rescue the phenotype and return SG numbers to those seen in non-AA-starved levels. In alignment with previous work demonstrating glutamine addiction in cancer cells (Reitzer et al., 1979), we confirmed that the effect was specific to cancer cells.

As cancer cells are also heavily reliant on glucose (Yang et al., 2022), elements of glucose metabolism were also examined. Even glutamine-recovered cells could not form SGs when glycolysis was blocked with the 2DG glucose analog. This demonstrates that although glutamine is being utilized effectively to support the TCA cycle, it alone cannot sufficiently support the metabolic needs of the cell. An ATP pathway analysis confirmed that glutamine was directed to increase mitochondrial ATP production. However, even with increased mitochondrial ATP generation, a second energy input from glucose was required. Blocking this energy supply sufficed to reduce ATP levels below a certain threshold, resulting in the prevention of SG formation and the promotion of cell death.

Glutamine is an efficient energy source. Therefore, it is not surprising that severely distressed cells displayed metabolic rewiring compared to arsenite-treated cells, to devote more resources to glutamine uptake. Mechanistically, this was linked to increased levels of *MYC* expression, which were quantified both using NGS and on the single RNA level. The amino-acid-starved cell groups displayed the highest levels of *MYC* expression, corresponding to the highest expression levels of glutamine uptake transporters, the lowest levels of glucose uptake transporters, and the lowest levels of glucose uptake. Of note, cells prioritize glutamine uptake (SLCA1 transporter) and utilization (MYC) even when no glutamine is present in the medium. We noted that *MYC* transcripts were recruited to the SGs, perhaps enabling these transcripts to escape degradation, as SGs have been shown to protect RNA (van Leeuwen et al., 2022), mainly via mRNP stabilization by SG proteins (Bley et al., 2015). Here, we have demonstrated that MYC is essential in the utilization of glutamine for SG formation and cell survival, given that cells that received a MYC inhibitor during treatment failed to form SGs and showed increased cell death. This metabolic rewiring during AA starvation testifies to the central role glutamine plays in cancer cell metabolism, for it is used not only for direct energy output, thereby impacting energy-dependent pathways, but also for increasing other antiporters that rely on glutamine, as well as potentially nucleic acid metabolism (Mazat and Ransac, 2019; Yoo et al., 2020), marked by elevated gene clusters for DNA and RNA processing, the latter perhaps having a direct effect on SG formation by supplying the cell with available RNA (Angel et al., 2024).

An NGS analysis painted a comprehensive picture of gene expression modulation after the administration of glutamine to the AA-starved cells. Cells that were AA-starved and VBL-treated were unable to form SGs. These cells exhibited high elevation in pathways related to cell death and inflammation, and downregulation of genes related to cell cycle, growth and metabolism. Of note, the dramatic change occurred after the addition of only one type of amino acid. Glutamine was capable of pushing the cell toward a pre-starved state, denoted by the increase in the expression of genes related to DNA processing, RNA processing, mitochondrial and AA metabolism and the stress response. This demonstrates how the cell contends with the stress, before succumbing to programmed cell death. SG formation is another element employed to mount a successful response. The addition of glutamine decreased the expression of mitophagy-related genes while upregulating mitochondrial transport genes. This finding helps explain the radical decrease in mitochondrial ATP seen in the starved cells, and the dramatic increase in the mitochondrial fraction in the glutamine-recovered treatments. Altogether, a myriad of pathways are affected by glutamine addition, each contributing to cell survival in its own way.

ΔΔG3BP1/2 cells shed light on the specific role of the SG in this stress response. Previous work has demonstrated changes in cell survival after exposure to cellular stresses in ΔΔG3BP1/2 cells (Aulas et al., 2018; Schwed-Gross et al., 2022; Szaflarski et al., 2016; Szczerba et al., 2023). In this study, measurements of cell viability of the various treatments confirmed that glutamine-recovered cells indeed show increased survival, and that starved cells that fail to form SGs die faster and in higher numbers. However, the images captured in WT cells revealed that SGs formed rapidly upon arsenite exposure and then dissolved after ∼1.5 h. Mechanistically, we believe this to be a crucial part of the observed survival. SGs form upon stress and are not meant to persist without a limit. When they dissolve, perhaps they release their cargo for translation. Additionally, SGs can sequester apoptosis and other pro-death factors from the bulk cytosol (Arimoto et al., 2008; Park et al., 2020). Not having a SG ‘buffer’ system in place in ΔΔG3BP1/2 cells, means that these cells cannot sequester apoptotic proteins (Fujikawa et al., 2023), which yields cell death. This was true also when the G3BP1/2 KO cells were enriched with glutamine.

A surprising insight came from the metabolic rewiring observed in ΔΔG3BP1/2 cells after arsenite exposure. Although the KO cells showed similar ATP levels to those of WT cells under untreated conditions, they exhibited enhanced glucose uptake in almost every treatment group, which corresponded with lower levels of *MYC*. Although glutamine did increase the overall glucose uptake, it did not improve the mitochondrial ATP fraction at all, indicating that glutamine was not being metabolized directly and was not supporting the TCA cycle. This was seen in the cell death assays as well. While the number of dead cells began to rise slowly at ∼7 h in the glutamine-recovered group, ΔΔG3BP1/2 cells began dying much earlier, at 4 h, despite the presence of glutamine. Not only did these cells lack SGs, but they also rewired their glutamine metabolism, demonstrating that glucose and glutamine are taken up in a G3BP1/2-dependent manner.

The cellular stress response is highly orchestrated and coordinated. It involves the halting and activation of a wealth of processes, the altering of metabolic pathways, as well as the formation of specialized structures. SGs have long been linked to a host of maladies and pathologies, and are here demonstrated to not only be integral to the cancer stress response, but also to be intricately connected to metabolic activity during stress.

## MATERIALS AND METHODS

### Cell culture and transfections

Human U2OS (ATCC) and U2OS ΔΔG3BP1/2 cells and G3BP1-rescued cells (Kedersha et al., 2016) (a gift from Nancy Kedersha and Pavel Ivanov, Brigham, and Women's Hospital and Harvard Medical School, Boston, MA, USA) as well as cells stably expressing GFP–IGF2BP3^33^ were maintained in low-glucose DMEM (01-050A Sautorios, Israel). A549 cells (a gift from Amit Tzur, Bar-Ilan University, Israel), MCF7 (ATCC), HeLa (ATCC) and HFF-1 (a gift from Ron Goldstein, Bar Ilan University, Israel) cells were grown on high-glucose DMEM (41965039, Gibco, USA). HCT116 cells (ATCC) were maintained in McCoy's 5A medium (Biological Industries). All cells were supplemented with 10% fetal bovine serum (SV30160.03, HyClone Laboratories, Logan, UT) and 100 IU/ml penicillin and 100 μg/ml streptomycin (Biological Industries, Israel). MCF10-A cells (a gift from Ofir Hakim, Bar Ilan University, Israel) were maintained in mammary epithelial cell medium (ScienCell, 7611).

Arsenite (0.25 mM; Sigma) was added to the medium for 50 min or was used at 0.0625 mM for 12 h. VBL (300 nM; Sigma) was added to the medium for 18–20 h before the arsenite treatment. For AA starvation, cells were washed twice with 1× PBS and incubated with EBSS medium without FBS (Biological Industries). For MYC inhibition, cells were treated with 40 µM of 10058-F4 (Merck F3680), for 18-20 h before arsenite treatment. For ATP synthase inhibition, cells were treated with 5 µM of oligomycin A (Sigma, 75351) for 20 min before arsenite treatment. For ER stress, cells were treated with 10 µM of thapsigargin (Sigma, T9033) for 1 h.

### Human primary tissue-derived organoids

For the establishment of human primary tissue-derived airway organoids, lung tissue was obtained via the Sheba Tissue Bank (Chaim Sheba Medical Center, Israel). Organoids were established and maintained according to a published protocol (Sachs et al., 2019). Briefly, fresh tissue was mechanically and enzymatically digested. Isolated cells were plated in adherent Cultrex growth-factor-reduced basement membrane extract (BME) type 2 drops and overlaid with an optimized organoid culture medium containing 10% R-spondin-1-conditioned medium produced from HEK293 HA–Rspo1–Fc cells (Cultrex^®^ HA–R-spondin-1–Fc 293T cells; 3710-001-01) and 10% Noggin-conditioned medium produced from HEK293 cells stably transfected with pcDNA3 NEO containing mouse Noggin insert (kindly provided by the Hubrecht Institute, The Netherlands). The medium was changed every 4 days, and organoids were passaged every ∼2 weeks using mechanical shearing with TrypLE Express (Invitrogen, 12605036).

The use of human tissue via Sheba Tissue Bank was approved by the local ethics committee and by the Associate Director at the Sheba Medical Center (approval no. 7188-20-smc), and informed consent was obtained from the tissue donor. Investigations were conducted according to the principles expressed in the Declaration of Helsinki.

For the immunostaining, whole organoids were suspended in BME, plated in an 18-well µ-slide (81816, ibidi) and covered with appropriate growth medium overnight or longer. Following the treatments, the organoids were fixed in 4% paraformaldehyde (PFA) for 30 min, permeabilized with 0.3% Triton X-100 in phosphate-buffered saline (PBS) for 30 min, and then blocked with 3% BSA in 0.01% Triton X-100 in PBS (PBST) for 1 h. The organoids were incubated with the primary antibody anti-G3BP1 (1:200, Abcam, ab56574) for 2 h at room temperature (RT), and then for 1 h at RT with the secondary antibody Cy3–Alexa Fluor goat anti-mouse IgG (1:1000, Abcam, ab97035) together with phalloidin–FITC (5 µM; Sigma). Antibodies were diluted in 3% BSA in PBST, and incubations were followed by three washes of 5 min with PBST. DNA staining was performed with Hoechst 33342 for 10 min, and organoids were then covered with PBS.

### Immunofluorescence

Cells were grown on coverslips in 12-well plates and fixed in 4% paraformaldehyde (PFA) for 20 min. Cells were permeabilized in 0.5% Triton X-100 for 2 min, and blocking was applied using 5% bovine serum albumin (BSA) fraction V (MP Biomedicals, 160069). Then, cells were incubated with primary antibodies (listed below) for 1 h, washed with 1×PBS, and incubated with secondary fluorescent antibodies (listed below). The nucleus was stained with Hoechst H33342 (1 μg/ml, Sigma, B2261) and coverslips were mounted in a mounting medium (homemade). Primary antibodies were: anti-G3BP1 (1:200, Abcam, ab56574), anti-Dcp1a (1:250, Abcam, ab183709) and anti-α-tubulin (1:400, Abcam, ab15568). Secondary antibodies were: Alexa Fluor 488-conjuated goat anti-mouse IgG (1:1000, Abcam, ab150113), Alexa Fluor 488-conjuated goat anti rabbit IgG (1:1000, Abcam, ab150077), Cy3–Alexa Fluor goat anti-mouse IgG (1:1000, Abcam, ab97035), Cy7–Alexa Fluor goat anti-mouse IgG (1:500, Abcam, ab175738), Alexa Fluor 647-conjuated anti-mouse IgG (1:1000, Invitrogen, A21235), 647-AlexaFluor anti-rabbit (1:1000, Invitrogen, A31573).

### SG correlation analysis

Image analysis was performed using a custom-made ImageJ macro (available upon request). The analysis pipeline incorporated standard image-processing techniques, such as adjusting contrast and brightness and filters to better distinguish the clusters, and utilized built-in Find Maxima and Particle Analysis algorithms in ImageJ to detect and characterize the clusters while filtering out noise. Further statistics with the cluster's characterization were conducted with the Seaborn library in Python (https://pypi.org/project/seaborn/) using a Welch's *t*-test.

### Fluorescence *in situ* hybridization

Cells were grown on coverslips and fixed in 4% PFA for 20 min, then kept in ethanol 70% (CARLO ERBA Reagents, 4146052) overnight at 4°C. RNA smFISH experiments with Stellaris Myc probes (Biosearch Technologies) were performed according to their adherent cell protocol. The probe set used was Quasar-570-labeled MYC (Ex 548/Em 566) at a concentration of 12.5 µM per coverslip.

### ATP assays

Colorimetric total ATP assays were performed according to the manufacturer's protocol (Abcam, ab83355). Seahorse XF Real Time ATP Rate assays were performed according to the manufacturer's protocol (Agilent Seahorse, 103592-100), with the use of Agilent Seahorse XF DMEM medium, pH 7.4 (Agilent Seahorse 103575-100).

### Glucose uptake assay

The assay relies on fluorescently labeled glucose 2-NBDG (Thermo Fisher Scientific, N13195) as an indicator for glucose uptake and on high-performance liquid chromatography (HPLC) for detection. First, the cells were incubated with the analog prior to liquid extraction. Next, cells were harvested with trypsin (Sigma), collected, centrifuged at 400 ***g*** for 5 min, and washed in cold 1×PBS. This process was repeated twice. Cells were then lysed in 100 µl 80% cold LC-MS methanol (Merck, 106035), and shaken at 40°C for 40 min at 600 rpm in a Thermal cycler (Eppendorf). Samples were next centrifuged at 20,000 ***g*** for 20 min and then 95 µl of the supernatant was transferred to a clean tube and centrifuged again at 20,000 ***g*** for 20 min while the pellets were taken for protein Bradford analysis for loading control. The supernatant was transferred to HPLC vials and analyzed using a Hitachi Elite LaChrom system (Hitachi, Tokyo, Japan) equipped with a fluorescence detector (L-2485), column oven, autosampler, quaternary pump and 100 μl sample loop. The chromatographic separation was performed at 30°C using an ACE 5 C-18 Avantor column (5 µm particle size, 4.6×250 mm) with a guard column cartridge of ACE 5 C-18 material under gradient elution conditions. Mobile phases (HPLC grade) consisting of water-trifluoroacetic acid (TFA; 1000:1, v/v) and acetonitrile-trifluoroacetic acid (1000:1, v/v). Gradient elution at a flow rate of 1.0 ml/min started at 3% B, increased linearly to 40% B for 9.5 min, and then increased linearly to 97% B until 10 min and then kept isocratic for an additional 5 min, until 15 min mark. For column equilibrium, the gradient was set back to 3% B until 15.5 and the system was allowed to equilibrate until 25 min. The injection volume was 25 μl, and the column elution was monitored at an excitation of 465 nm and emission of 540 nm. The chromatogram showed a peak of fluorescence at a retention time of 9.3 min, which was used to measure 2-NBDG for all samples, according to the 2-NBDG standard compound chromatogram. Chromatogram peak integration and area under the curve (AUC) calculations were performed using EZChrom Elite Software. AUC was normalized to protein amount. Cell amount and time of incubation with 2-NBDG were experimentally determined.

### RNA-seq

For library preparation, a Ribo-Zero Plus rRNA Depletion Kit (Illumina) was used from 1 μg of starting material. All RNA samples underwent rRNA depletion following the manufacturer's protocol. The libraries were amplified by ten PCR cycles in the PCR step. The quantification and quality control of the libraries were performed using a Denovix fluorometer and an Agilent 4200 TapeStation. Single-read sequencing of the libraries with a read length of 80 was performed with the NextSeq 500 Sequencing System using NextSeq 500/550 High Output v2 kit (75 cycles) (20024906 Illumina), yielding about 45 million reads per sample.

For bioinformatics analysis, sequenced reads were mapped to the human reference genome sequences (hg38) using STAR. The aligned reads were quantitated by Htseq. The normalization and differentially expressed genes test were implemented by DESeq2. An arbitrary cutoff of at least 1.5-fold and a *P*-value adjusted for multiple testing <0.05 were chosen to define a differentially expressed gene. The Metascape tool (Zhou et al., 2019) was used for enrichment analysis on the differentially expressed genes between each group. Gene expression heatmap was composed using Metaboanalyst 6.0 (Pang et al., 2024). Gene interaction map and cluster K-cluster analysis was performed using STRING tool (Szklarczyk et al., 2023). The RNA-seq data were deposited in NCBI GEO repository (GEO: GSE234436). All GO network references appear in Tables S2–S4. The Gene Set Enrichment Analysis (GSEA) was performed using the Molecular Signatures Database (MSigDB v7.5.1, http://www.gsea-msigdb.org/gsea/msigdb) to identify significantly enriched pathways and gene sets, with a false discovery rate (FDR) q-value<0.25 considered statistically significant.

### Cell death assay

Cell death was quantified using the specialized Incucyte Cytotox NIR Dye (Sartorius, 4846). Arsenite (0.625 µM) and Cytotox dye (0.6 µM) were added to each well before imaging. Cells were imaged every 30 min in the Incucyte SX5 specialized incubator and microscope imaging system with the SX5 G/O/NIR optical module.

### Imaging

Wide-field fluorescence images were obtained using the CellSens system based on an Olympus IX83 fully motorized inverted microscope (60× UPlanXApo objective, 1.42 NA) fitted with a Prime BSI sCMOS camera (Teledyne) driven by the CellSens software. Colocalization analysis of two channels was performed using an ImageJ macro (Shav-Tal lab, BIU). SG area was analyzed using ImageJ. Live-cell imaging was carried out using the CellSense system with rapid wavelength switching. Cells were plated on glass-bottomed tissue culture plates (MatTek, Ashland, MA, USA) in medium containing 10% FBS. Imaging was carried out at 37°C, using an incubator that includes temperature and CO_2_ control (Life Imaging Services, Reinach, Switzerland). Live-cell movies were edited by ImageJ. Long-term imaging and analysis was done using the Incucyte system (Sartorius) with a 20× objective lens.

### Western blotting

Cells were washed in cold PBS, and proteins were extracted using immunoprecipitation (IP) lysis buffer (Thermo Fisher Scientific) containing 10 mM NaF (Sigma), 10 mM sodium orthovanadate (Sigma), 1 mM protease inhibitor cocktail (Sigma) and 1 mM PMSF (Sigma). The samples were then placed on ice for 20–25 min. The resulting lysate was centrifuged at 14,000 rpm (20,817 ***g***) for 10 min at 4°C. Then, 20–40 µg/µl of protein per lane was run on SDS-polyacrylamide gels and transferred to a nitrocellulose membrane (0.45 µm; Bio-Rad). The membrane was blocked in 5% BSA for 1 h at RT or overnight at 4°C, and then probed with a primary antibody for 2 h at RT followed by incubation with an HRP-conjugated goat anti-rabbit IgG (Abcam, ab7090) or goat anti-mouse IgG secondary antibody for 1 h at RT. For loading control, the membranes were reprobed with an anti-α-tubulin or an anti-β-actin antibody. Immunoreactive bands were detected using an enhanced chemiluminescence kit (ECL, Pierce). Antibodies used were anti-GLUT1 (ab40084; 1:1000), anti-Myc (9E10, SC-40; 1:1000), anti-SLCA15 (ab237704; 1:1000), anti-β-actin (Cell Signaling 4970L; 1:1000) and rabbit anti-α-tubulin (Abcam, ab4074; 1:000). Experiments were performed at least three times. To normalize the different bands to control conditions and obtain a relative value, the ratio of protein was calculated to the untreated group. Quantification of band intensity from the blots was done using ImageJ software (NIH, Bethesda, MD). Uncropped images of blots shown can be found in Fig. S8.

### Luciferase assay

Luciferase assays were performed in U2OS cells. 2.5×10^4^ cells were plated in 96-well plates 24 h prior to transfection. Cells were transfected with a MYC-binding site plasmid, containing four E-box regions (100 ng) and 50 ng of Renilla Luciferase pLR-TK vector (Promega). Transfection was performed using the PolyJet transfection reagent (SignaGen, SL100688) at 3:1 PolyJet: DNA ratio. Luciferase activity was measured after 24 h using the Dual-Glo^®^ Luciferase Assay System according to the manufacturer's instructions (Promega, E2980). Results were normalized to *Renilla* expression and data were then represented according to untreated Luciferase expression. Statistical analyses were carried out using the Prism 9 software (GraphPad). Two-way ANOVA with the indicated post test was performed to compare samples.

## Supplementary Material



10.1242/joces.263679_sup1 Supplementary information

Table S2. Gene networks altered by starvation. GO details of networks altered in EBSS+VBL with arsenite treatment, compared to arsenite treated cells

Table S3. Heatmap of gene networks after glutamine recovery. GO details of the 1000 most altered networks in EBSS+VBL+ Glutamine with arsenite treated cells compared to cells that did not receive glutamine recovery.

Table S4. STRING network clusters after glutamine recovery. GO details of STRING network clusters altered in EBSS+VBL+ Glutamine with arsenite treated cells compared to cells that did not receive glutamine recovery.
