# Supplementary material for: Glutamine modulates stress granule formation in cancer cells through core RNA-binding proteins
Source: J Cell Sci. 2025 Jun 6;138(11):jcs263679. doi: 10.1242/jcs.263679 (PMC12188316; doi:10.1242/jcs.263679)
Supplement: Supplementary information [file joces-138-263679-s1.pdf]

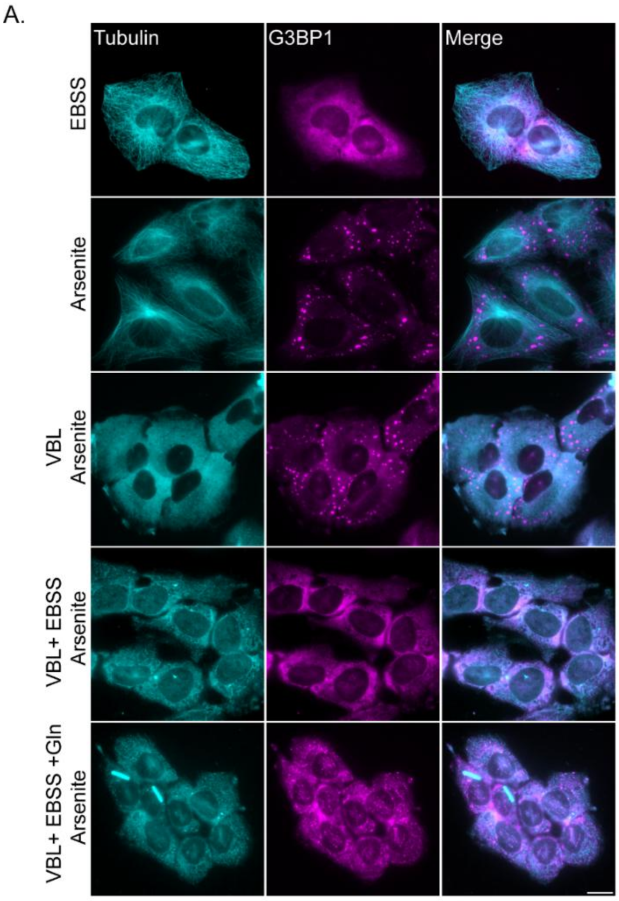

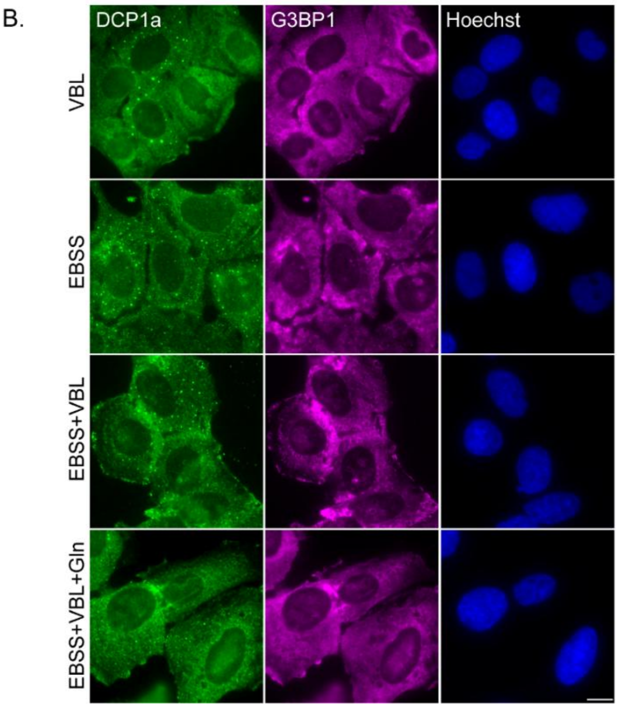

Journal of Cell Science • Supplementary information

Fig. S1. Examining the effects of treatments on cytoskeletal integrity and SG formation. **(A)** U2OS cells were stained by immunofluorescence with anti-α tubulin as a microtubule marker (green) and anti-G3BP1 as a SG marker (magenta). VBL dismantled MTs, while arsenite treatment did not. VBL could re-polymerize free tubulin into paracrystals (rod-shaped structures). Hoechst 33342 DNA stain is shown in blue. Scale bar, 20 µm. **(B)** U2OS cells stained by immunofluorescence with anti-Dcp1a as a PB marker (green) and anti-G3BP1 as a SG marker (magenta) show no SGs forming without the addition of arsenite. Hoechst 33342 DNA stain is shown in blue. Scale bar,20 µm.

Journal of Cell Science • Supplementary information

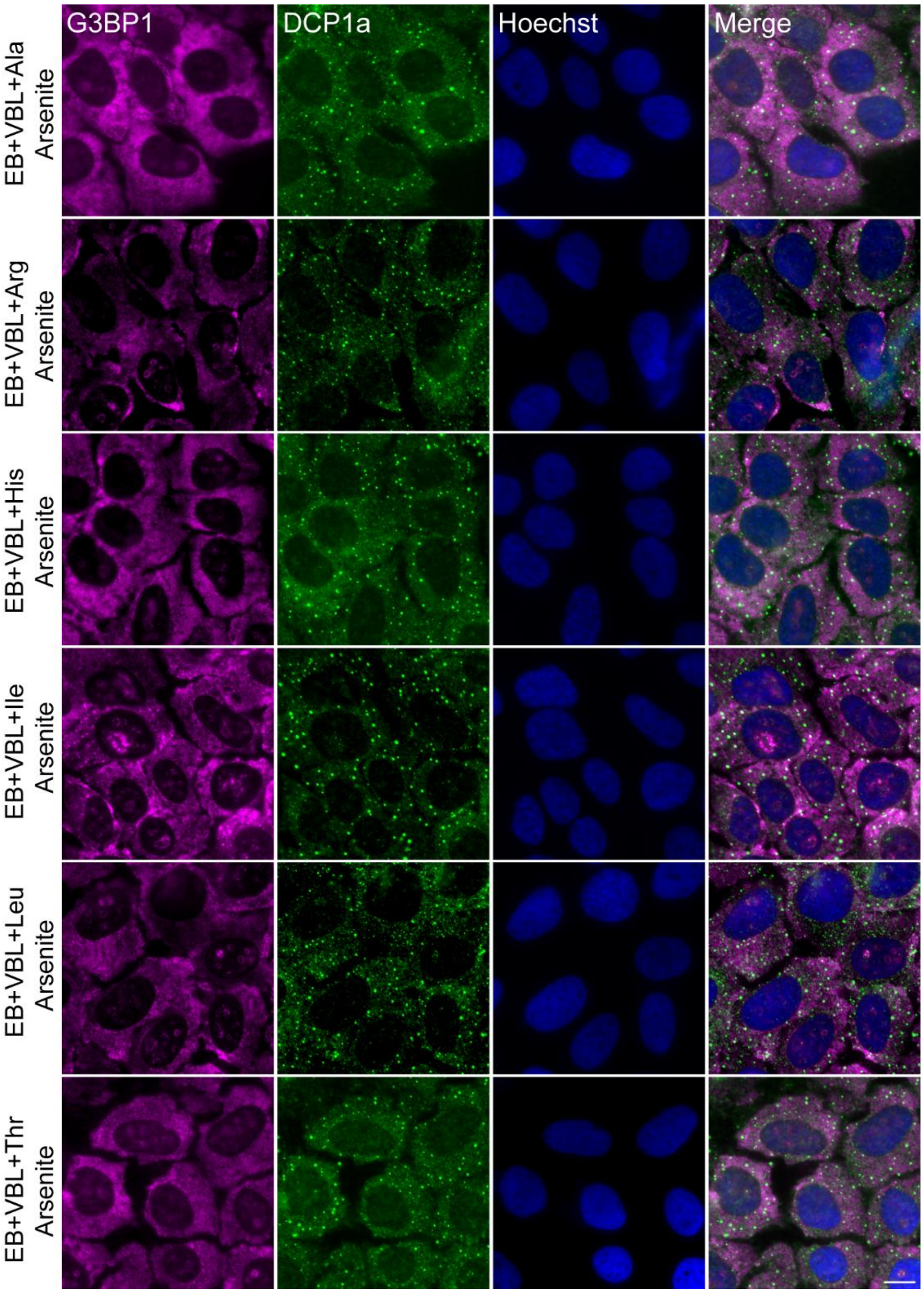

Fig. S2. **Screening the effects of various AAs on SG formation in starved cells**. U2OS cells stained by immunofluorescence with anti-G3BP1 as a SG marker (magenta) and anti-Dcp1a as a PB marker (green). Cells treated with AA-starvation (EB, EBSS) + VBL before arsenite treatment, with a single AA added to the cell culture. None of the screened AAs yielded SG formation. Hoechst 33342 DNA stain is shown in blue. Scale bar, 20 µm.

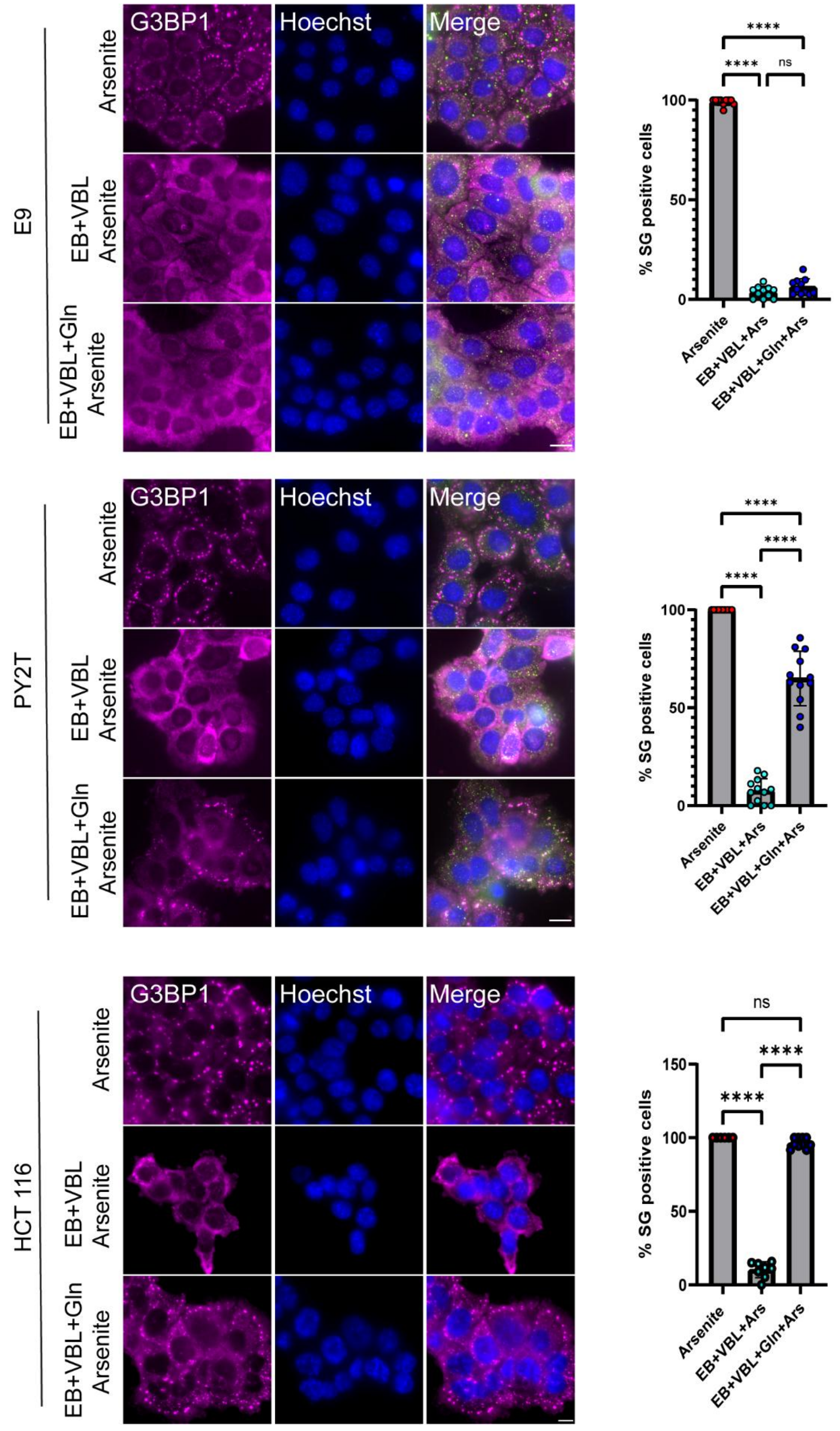

Journal of Cell Science • Supplementary information

Fig. S3. Screening for SG formation in various cell lines after glutamine supplementation. Additional screened cell lines under various treatments were stained with anti-G3BP1 as a SG marker (magenta) and anti-Dcp1a as a PB marker (green). E9 mouse epithelial cells, along with PY2T mouse cancerous epithelial cells both originating from breast/mammary gland were examined under various treatments. In each case, AA-starved (EB) + VBL treated cells before arsenite induction failed to form SGs. When glutamine was added to the starved cells, cancerous cells could utilize glutamine to form SGs, while non-cancerous cells could not. HCT116 were also subjected to the same treatments, and the cancerous cell line rescued the SG phenotype after glutamine supplementation. Hoechst 33342 DNA stain is shown in blue. Scale bar, 20 µm. For quantifications of the cell population positive to SGs, data were analyzed using one-way ANOVA (ns= nonsignificant, ****P*<0.001, *****P*<0.0001).

Journal of Cell Science • Supplementary information

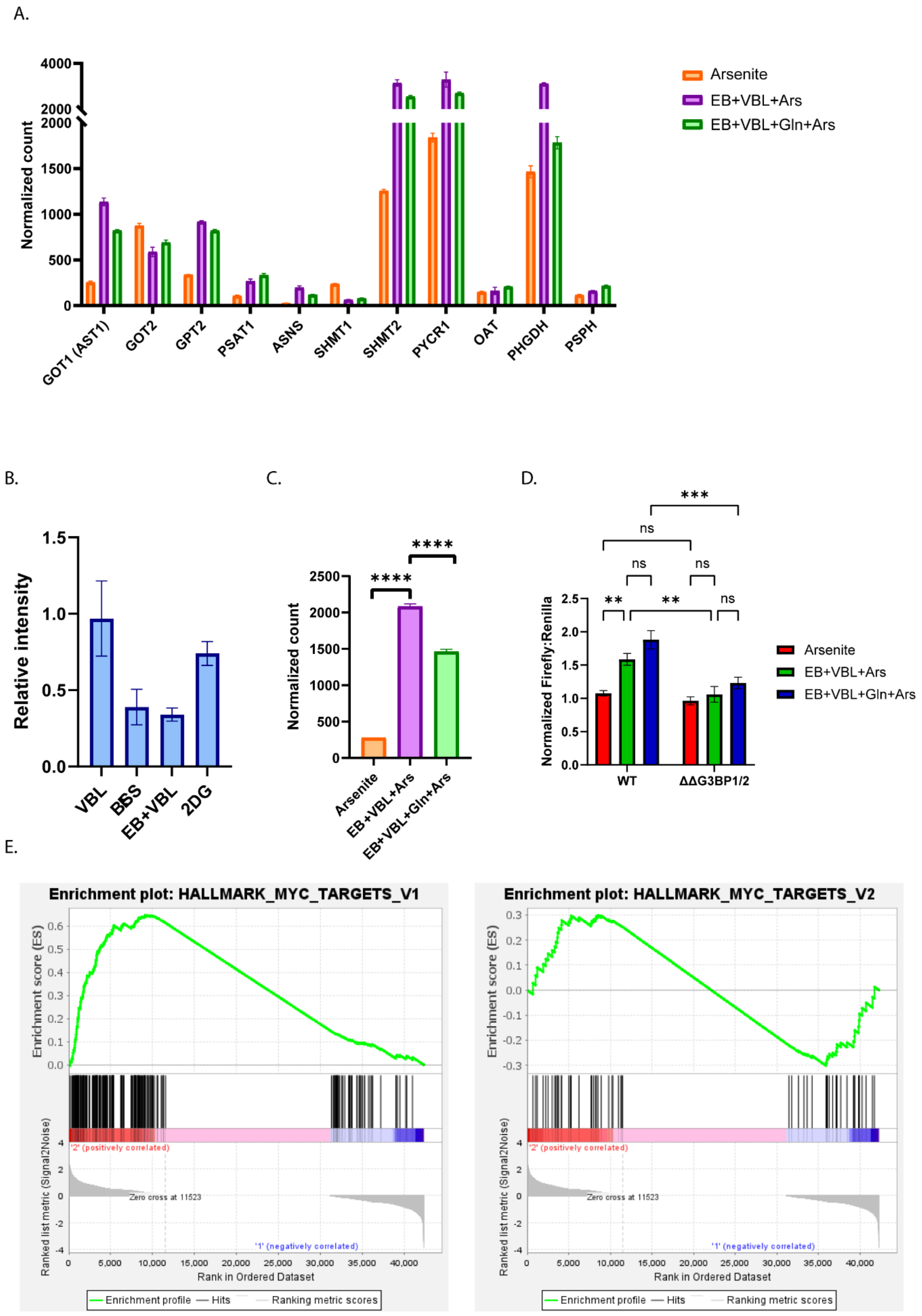

Journal of Cell Science • Supplementary information

Fig. S4. Measuring gene expression levels of genes involved in metabolic mechanisms. (A) NGS normalized counts of multiple genes involved in AA synthesis. Most genes showed an increase after AA-starvation +VBL with and without glutamine, indicating cellular response to severe lack of available AAs. **(B)** ATP measurements under the different treatment conditions. ATP measurements here are only slightly elevated compared to treatment groups that were also administered arsenite. **(C)** NGS counts of *MYC* transcripts under various treatments. Data analyzed using one-way ANOVA (*****P*<0.0001). (**D**) Luciferase assay with an E-box MYC binding site repeat sequence. EB+VBL and glutamine supplemented groups show increased MYC binding activity, while G3BP1/2 knock-out cells (DKO) show little increased activity across treatments. Data analyzed using one-way ANOVA (***P*<0.01). **(E)** GSEA enrichment plots showing MYC target sets upregulated when comparing AA starved (EB) +VBL groups to those that received the glutamine supplement. While each set shows elevated MYC gene expression and increased MYC target sets, one prioritizes 'V1' (EB+VBL+Gln+Ars, left), while the other 'V2' (EB+VBL+Ars, right).

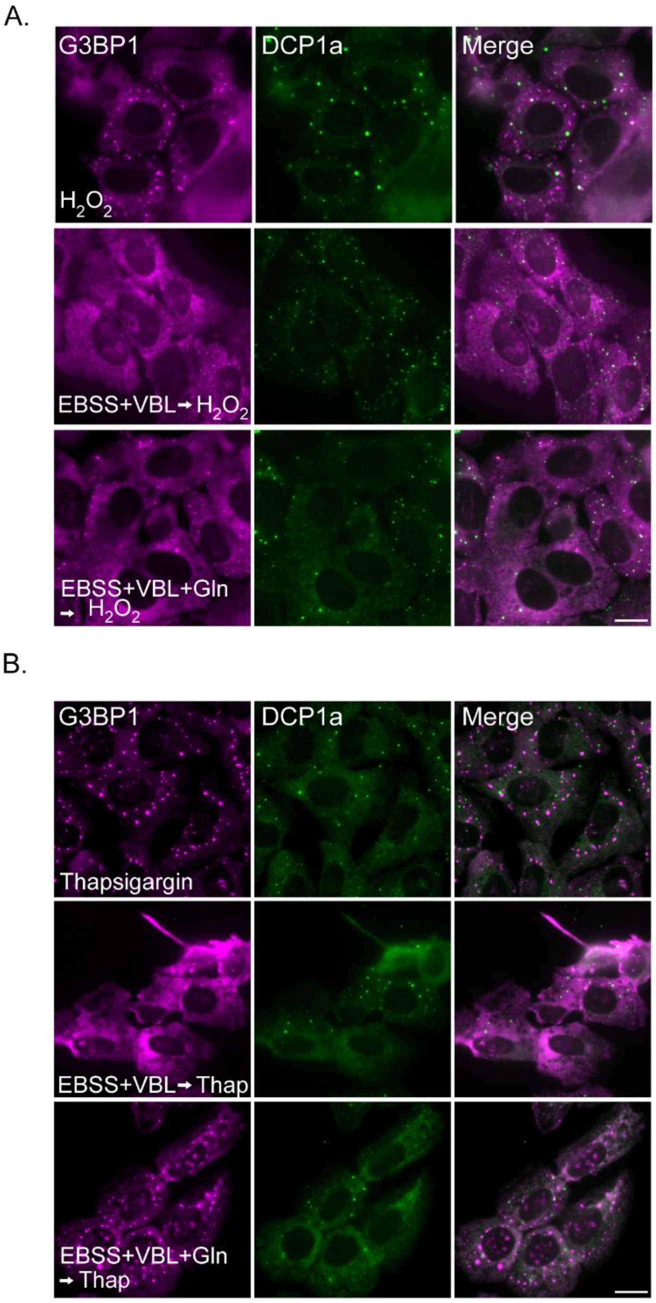

Fig. S5. The response of U2OS to other stressors. U2OS cells stained by immunofluorescence with anti-G3BP1 as a SG marker (magenta) and anti-Dcp1a as a PB marker (green). Cells treated with **(A)** hydrogen peroxide (H_2O_2), with AA-starvation (EB, EBSS) + VBL before treatment, or with glutamine added to the cell culture. **(B)** Cells were treated with the ER stressor thapsigargin, along with the same starvations and glutamine addition. Scale bar, 20 μm.

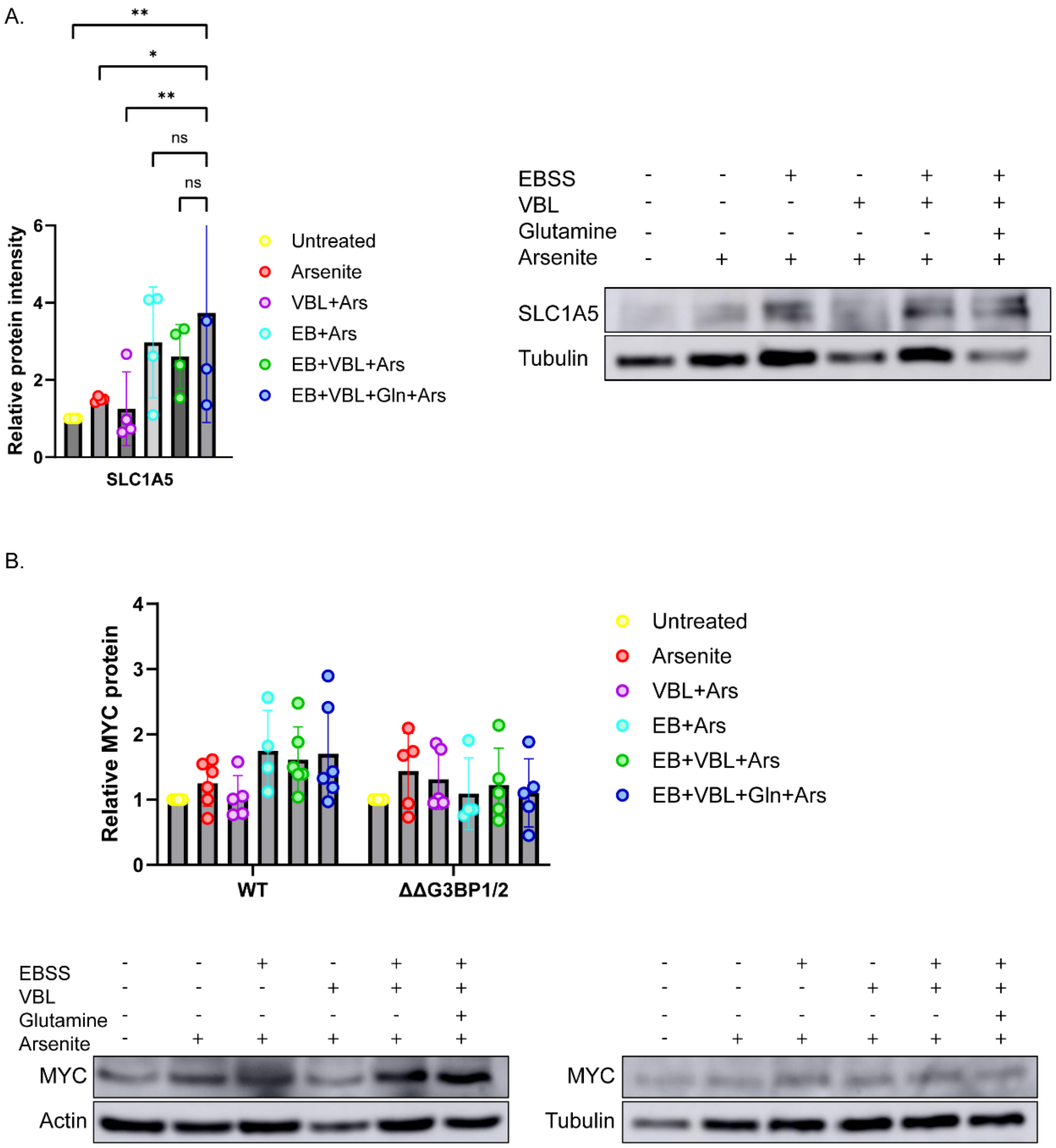

Fig. S6. Western blot analysis for proteins involved in the response to starvation. (**A**) Western blot quantification of the SLC1A5 glutamine transporter. All cell groups exposed to chronic AA starvation show elevation of the transporter. (**B**) Western blot quantification of the MYC protein in WT (left blots) and G3BP1/2 KO (right blots) cells. AA-starved cell groups show a mild increase in MYC expression, while KO cells do not show any change after chronic starvation or glutamine supplement.

Journal of Cell Science • Supplementary information

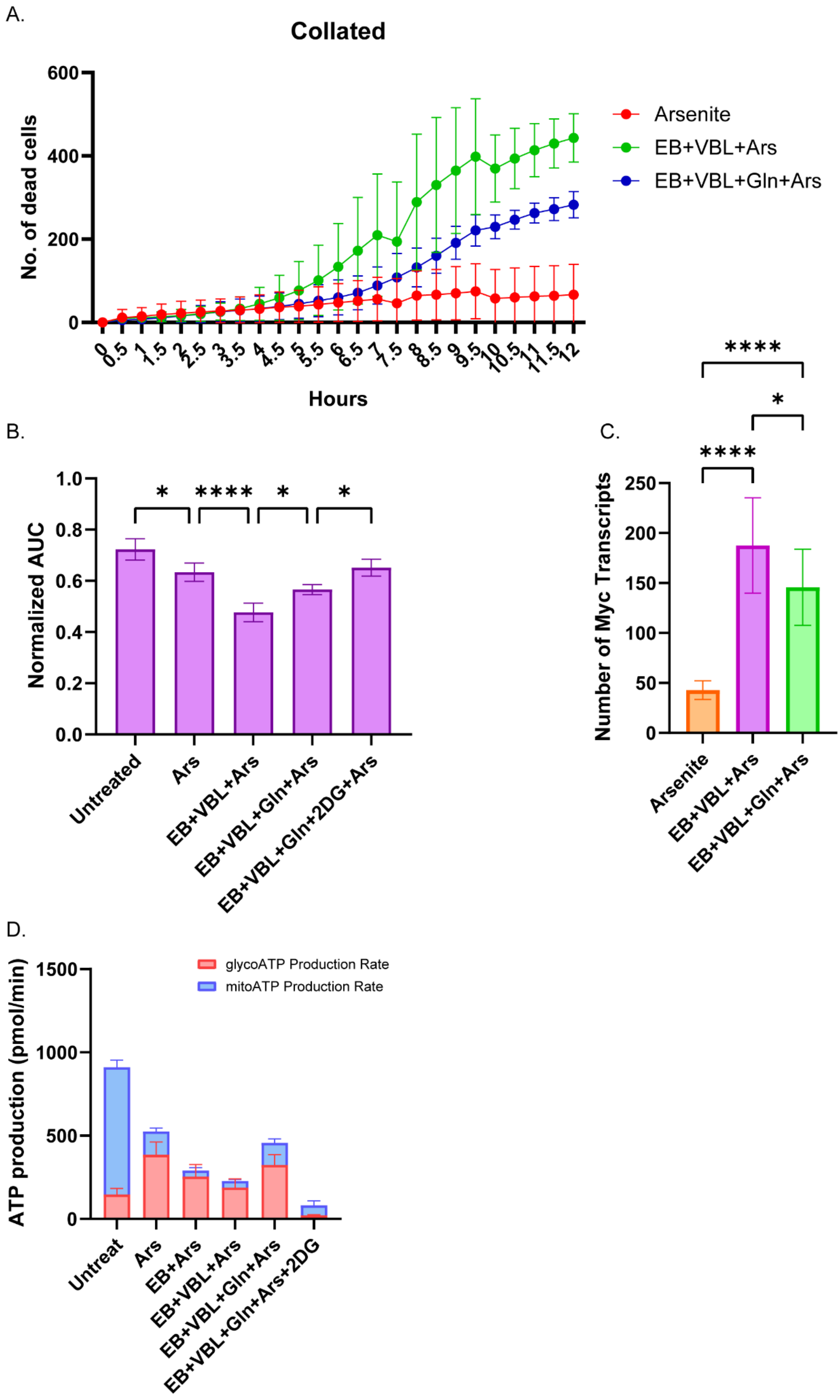

Journal of Cell Science • Supplementary information

Fig. S7. GFP-G3BP1 genetic rescue of core SG protein after knockout. (A) Quantifications of dead cells over time measured by the Cytotox NIR dye under various treatments and arsenite exposure (0.625 µM) in ΔΔG3BP1/2 U2OS cells stably expressing GFP-G3BP1. Cells after genetic rescue show similar cell death to WT cells (n=4). **(B)** NBDG glucose uptake assay in ΔΔG3BP1/2 cells expressing GFP-G3BP1. Glucose uptake is somewhat elevated compared to WT cells, but similar to WT cells demonstrate a significant reduction after arsenite and less glucose uptake compared to ΔΔG3BP1/2 cells. Data analyzed using one-way ANOVA (*P<0.05, ****P<0.0001). **(C)** Quantification of *MYC* transcripts using smRNA FISH in ΔΔG3BP1/2 cells expressing GFP-G3BP1. Rescue cells show a similar pattern of expression to WT cells. **(D)** Seahorse XF ATP rate assay for quantifying ATP and dividing into glycolytic (red) and mitochondrial (blue) fractions in ΔΔG3BP1/2 cells expressing GFP-G3BP1. Rescue cells show a similar pattern to WT cells, and show an increase in mitochondrial ATP after glutamine addition, something not seen in ΔΔG3BP1/2 cells.

Journal of Cell Science • Supplementary information

MYC-WT

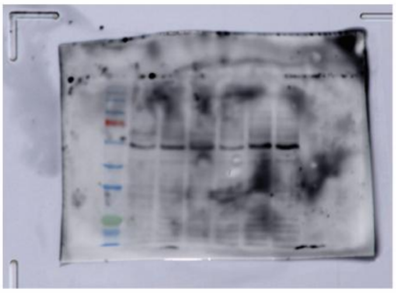

MYC-WT- Actin loading control

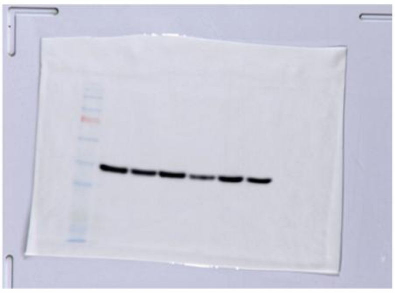

SLC1A5-WT

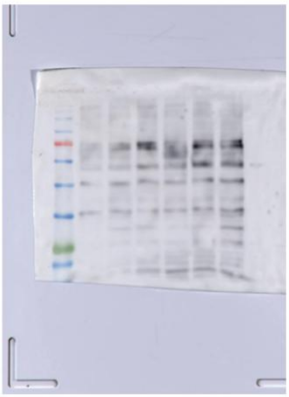

SLC1A5-WT Tubulin loading control

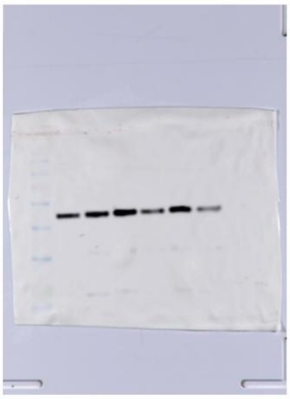

G3BP1- DKO MYC

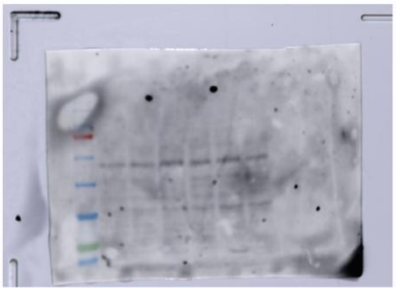

G3BP1-DKO Tubulin loading control

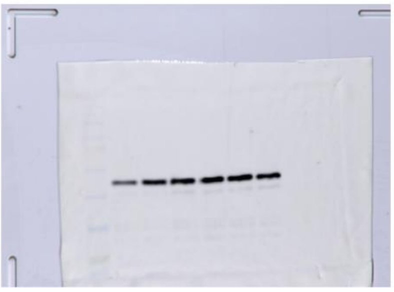

Fig. S8. Original western blot images for blot transparency

Journal of Cell Science • Supplementary information

Table S1. (A) Overview of effect of treatments on SG morphology. **(B)** Outline of treatment durations and order of administration.

A.

Treatment	Description	Effect on SGs
Arsenite	Induces oxidative stress	Forms large SGs
EBSS	AA and nutrient deprived medium	Limits SG number but does not prevent
VBL	Disrupts MT network	Induces many scattered and small SGs
EBSS+VBL	-	Inhibits SG formation
Glutamine (Gln)	AA, upon which cancer is heavily reliant	When added to EBSS+VBL treated cells rescues SGs
2-Deoxy-Glucose (2DG)	ATP inhibitor, nonfunctional glucose analogue	Blocks any recovery form glutamine rescue

B.

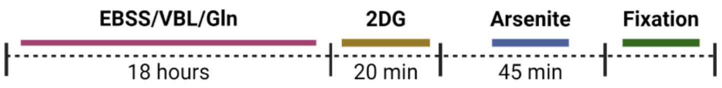

Table S2. Gene networks altered by starvation. GO details of networks altered in EBSS+VBL with arsenite treatment, compared to arsenite treated cells

Available for download at
https://journals.biologists.com/jcs/article-lookup/doi/10.1242/jcs.263679#supplementary-data

Journal of Cell Science • Supplementary information

Table S3. Heatmap of gene networks after glutamine recovery. GO details of the 1000 most altered networks in EBSS+VBL+ Glutamine with arsenite treated cells compared to cells that did not receive glutamine recovery.

Available for download at
https://journals.biologists.com/jcs/article-lookup/doi/10.1242/jcs.263679#supplementary-data

Table S4. STRING network clusters after glutamine recovery. GO details of STRING network clusters altered in EBSS+VBL+ Glutamine with arsenite treated cells compared to cells that did not receive glutamine recovery.

Available for download at
https://journals.biologists.com/jcs/article-lookup/doi/10.1242/jcs.263679#supplementary-data

Journal of Cell Science • Supplementary information

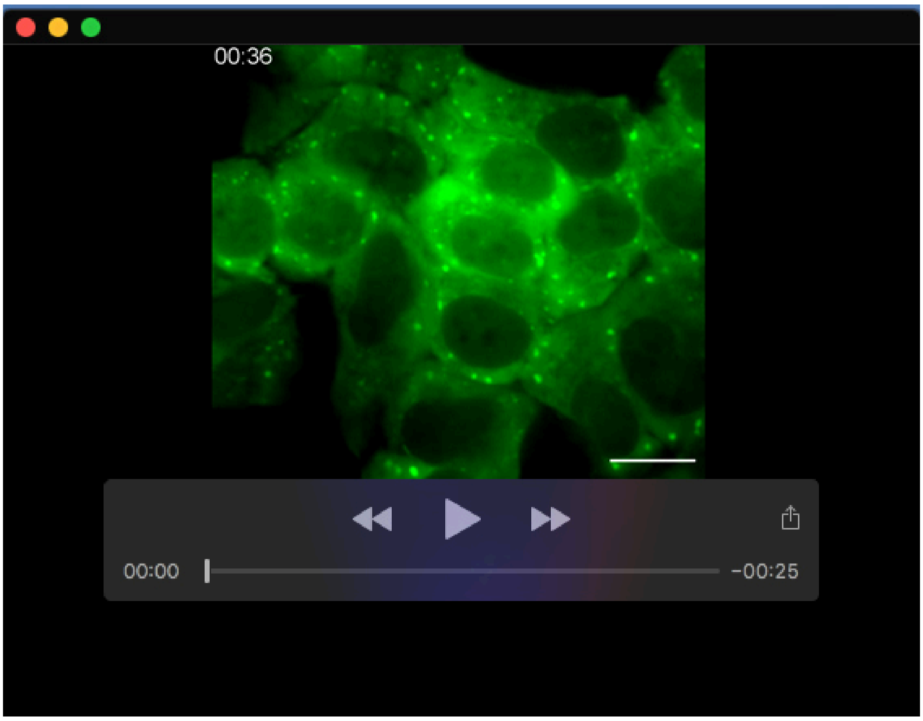

Movie 1. Live cell imaging of SG formation under arsenite treatment. Movie of GFP-IGF2BP3 expressing U2OS cells imaged for 68 min every 4 min showing the formation of SG-positive cells over time during arsenite treatment. Most cells form SGs. Bar 20 µm.

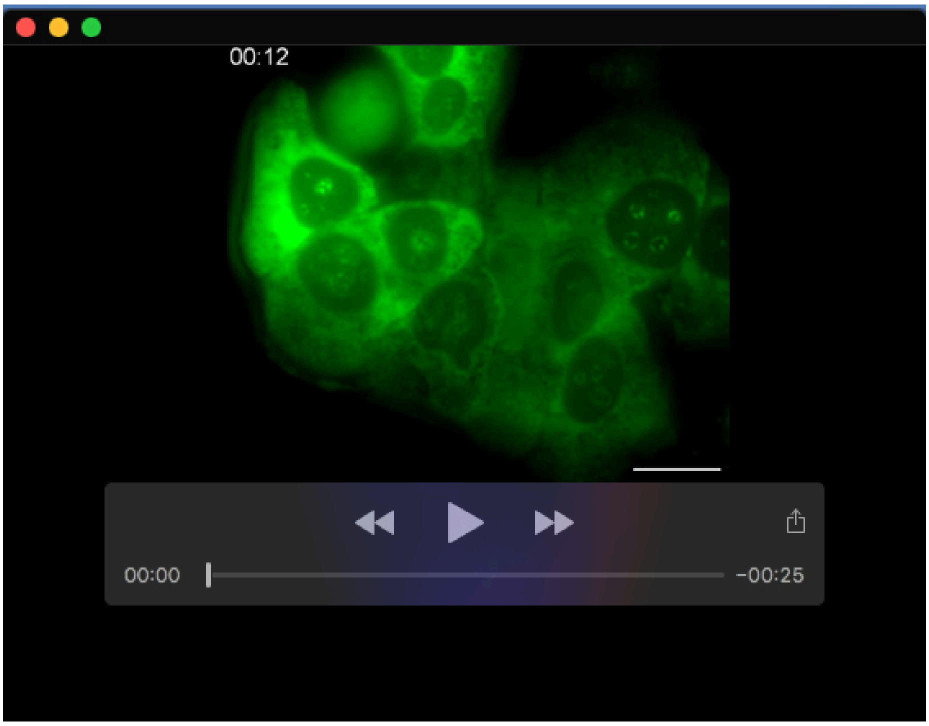

Movie 2. Live cell imaging of SG formation under amino acid starvation and arsenite treatment. Movie of GFP-IGF2BP3 expressing U2OS cells imaged for 68 min every 4 min showing the formation of SG-positive cells over time in cells treated with amino acid starvation (EBSS)+VBL followed by arsenite. Very few cells succeed in forming SGs. Bar 20 µm.

Journal of Cell Science • Supplementary information

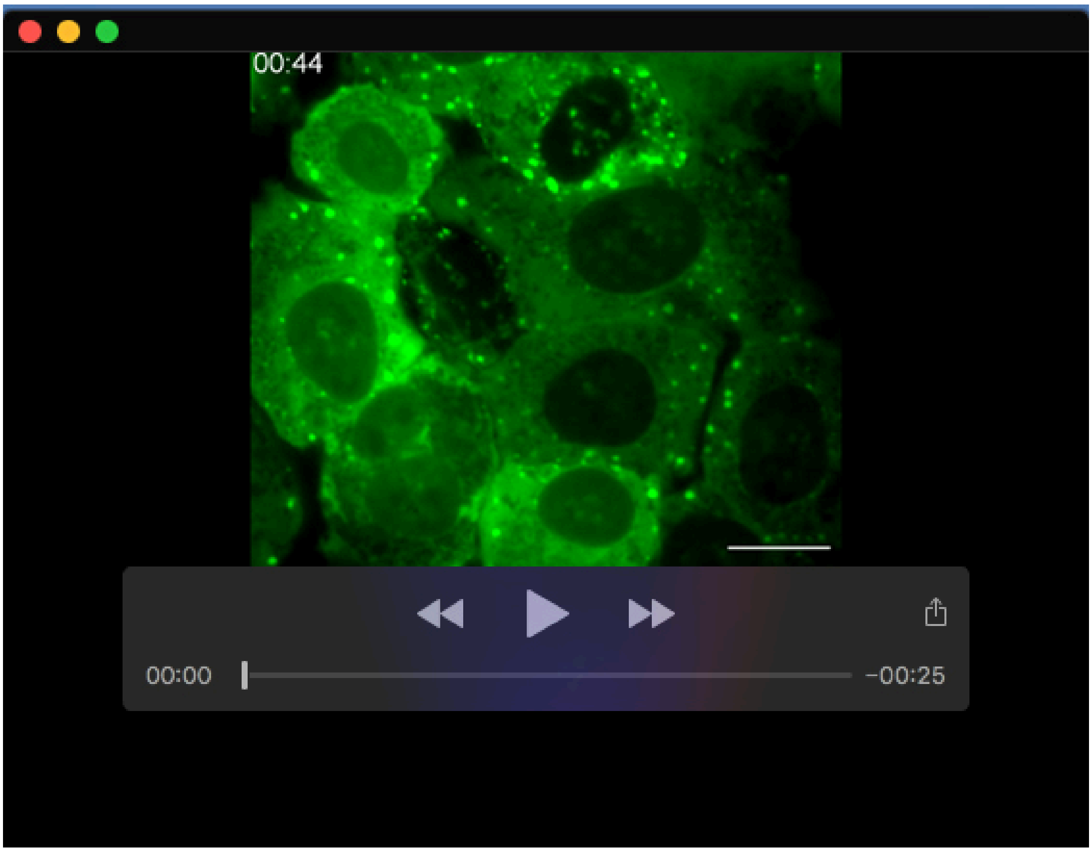

Movie 3. Live cell imaging of SG formation under glutamine recovery and arsenite treatment. Movie of GFP-IGF2BP3 expressing U2OS cells imaged for 68 min every 4 min showing the formation of SG-positive cells over time in cells treated with amino acid starvation (EBSS)+VBL and glutamine followed by arsenite. Most cells form SGs. Bar 20 µm.
