# Supplementary material for: Glutamine modulates stress granule formation in cancer cells through core RNA-binding proteins
Source: J Cell Sci. 2025 Jun 6;138(11):jcs263679. doi: 10.1242/jcs.263679 (PMC12188316; doi:10.1242/jcs.263679)
Supplement: Supplementary file 1 [file joces-138-263679_review_history.pdf]

Glutamine modulates stress granule formation in cancer cells through core RNA-binding proteins

Gabriel P. Faber, Gross Gilad, Oz Mualem, Matan Y. Avivi, Hiba Waldman Ben-Asher, Orly Yaron, Noa Kinor, Orit Shefi, Rakefet Ben-Yishay, Dana Ishay-Ronen and Yaron Shav-Tal
DOI: 10.1242/jcs.263679

Editor: Daniel Billadeau

Review timeline
Original submission: 31 October 2024
Editorial decision: 2 December 2024
First revision received: 3 March 2025
Accepted: 25 April 2025

Original submission

First decision letter

MS ID#: jcs.263679

MS TITLE: Glutamine modulates stress granule formation in cancer cells through core RNA-binding proteins

AUTHORS: Yaron Shav-Tal; Gabriel P Faber; Gross Gilad; Oz Mualem; Matan Y Avivi; Hiba Waldman Ben-Asher; Orly Yaron; Orit Shefi; Rakefet Ben-Yishay; Dana Ishay-Ronen

ARTICLE TYPE: Research Article

Dear Dr Shav-Tal,

We have now reached a decision on the above manuscript.

To see the reviewers' reports and a copy of this decision letter, please go to:

As you will see, the reviewers raise several criticisms that prevent me from accepting the paper at this stage. They suggest, however, that a revised version might prove acceptable, if you can address their concerns. If you think that you can deal satisfactorily with the criticisms on revision, I would be pleased to see a revised manuscript. We would then return it to the reviewers.

Reviewer 1

Advance summary and potential significance to field

In their study, Faber et al. report very interesting findings on the energy level dependent formation of stress granules. The authors demonstrate a strong impact of glutamine and glucose metabolism on the formation of stress granules (SGs), which to the best of my knowledge have not been reported to this level so far. Based on their findings they claim that a certain energy threshold is required to permit stress granule formation and that this is largely influenced by the master oncogene MYC. These observations could potentially have a great impact on our understanding of

© 2025. Published by The Company of Biologists under the terms of the Creative Commons Attribution License (https://creativecommons.org/licenses/by/4.0/).

regulatory mechanisms of SG homeostasis in cancer cells, how this is linked to metabolic deregulation in cancer, and may even lead to novel therapeutic vulnerabilities.
The presented findings are largely supported by the provided data, although they remain largely descriptive. Nonetheless, the authors need to provide some additional controls to solidify their findings, specifically when using inhibitors, for instance of MYC. Likewise, it remains debatable if changes in mRNA levels over comparatively short perturbation time frames are key indicators of altered pathway activities. To this end, the authors should consider evaluating protein levels and gene expression data by GSEA.

Comments for the author

1. If the glutamine-dependency in SG formation is largely cancer cell specific, is it related to altered KRAS mutational status? This was reported to impact on the glutamine addiction of cancer cells. Although the authors provide evidence of conservation in A549 and MCF7 cells, it would be interesting to consider this aspect in additional cancer cell models with and without KRAS mutations.
2. In respect to the energy dependency, it would be informative to evaluate if other stressors leading to specific ATP depletion, for instance by the FDA-approved drugs targeting ATP5F1C recapitulates the presented observations.
3. The analyses in Fig.4 certainly support the authors conclusion that SGs operate in a larger context, in particular energy production. However, arsenite likely induces a largely energy-related stress affecting ATP production via mitochondria, as indicated also indicated in the manuscript. Thus, the authors should consider testing other stress inductions, e.g. ER, heat and/or hypoxic stress, to test if glutamine/Glu are equally important in such scenarios.
4. In respect to gene expression analyses, the authors should deepen their evaluation of NGS data by GSEA analyses to get more insights on altered pathway activities, e.g. MYC-driven transcription etc.
5. The findings of altered glutamine transport capacities require further validation. The authors should validate their findings on the protein level, since an adaption of mRNA levels may not properly reflect changes of transport capacity at the plasma membrane. Furthermore, how do the authors envision that stress induction promotes such changes in gene expression - at the transcriptional level or altered RNA turnover? Overall translation should be largely silenced in stressed cells?
6. This referee is not an expert in 2DG-directed block of glycolysis and glycolysis-dependent ATP production. How can the authors exclude that 2DG also affect mitochondrial production of ATP and how can they potentially solidify their findings by additional controls or alternative inhibitors.
7. The findings on MYC are interesting. However, it remains to be clarified if changes in MYC act mainly via transcriptional regulation, for instance by monitoring MYC transcriptional reporter activity. In addition, it remains unclear if changes in MYC abundance are also seen on the protein level and if they result from altered transcription, mRNA or protein turnover. This should be addressed in view of the rather short perturbation times. Finally, the authors should control for MYCi and should investigate their gene expression data (Fig.4) for alterations in MYC pathways, for instance via GSEA studies (see above).
8. The observations in G3BP1/2-KO cells are interesting but would benefit from further in-depth testing of the MYC connection and altered gene expression levels. To this end, the authors should consider evaluating gene expression profiles by NGS and/or monitor MYC abundance at the protein level and its transcriptional activity by luciferase reporters.
9. Minor comment: The investigated glutamine transporters in Fig.5C do not cover all glutamine transporters reported in mammalian cells, especially they lack SLC7A5 (LAT1), which is considered major to my knowledge. Could the authors comment on their selection and extend their analysis if appropriate?

Reviewer 2

Advance summary and potential significance to field

This is well-developed work. The results are interesting and novel. The finding that the sole amino acid source governing whether cancer cells form SGs during stress or not is of particular note. In

© 2025. Published by The Company of Biologists under the terms of the Creative Commons Attribution License (https://creativecommons.org/licenses/by/4.0/).

agreement with it, metabolic profiling revealed the essential functions of glutamine and glucose in SG formation under limiting metabolic conditions. Interestingly, inhibition of MYC prevented SG formation, which is important finding of the work.

I only have few minor suggestions to improve the work.

Comments for the author

1. Myc levels are determined on mRNA level. Authors should back up their myc mRNA changes by western blotting detecting Myc protein. I think the fact that Myc mRNA is found to be localized into SG may change its availability to translation and thus limit Myc protein production
2. Core binding proteins. Authors should use siRNA to KD other "core" Sg proteins that are influencing SG formation, e.g. UBAP2L and/or DDX6
3. How does overexpression of G3BP affect SG behavior? Will it overcome metabolic "restrictions" for SG dynamics? Will it affect cell viability and survival?

First revision

Author response to reviewers' comments

We thank the reviewers for their insightful comments. Following are our responses describing how we implemented the suggestions and requests, which we have tried to address in a comprehensive manner. Part of the experiments and data were added to the manuscript and some serve here in the letter as a specific response to points raised. We believe the manuscript has improved thanks to the reviewers careful reading.

Reviewer 1: In their study, Faber et al. report very interesting findings on the energy level dependent formation of stress granules. The authors demonstrate a strong impact of glutamine and glucose metabolism on the formation of stress granules (SGs), which to the best of my knowledge have not been reported to this level so far. Based on their findings they claim that a certain energy threshold is required to permit stress granule formation and that this is largely influenced by the master oncogene MYC. These observations could potentially have a great impact on our understanding of regulatory mechanisms of SG homeostasis in cancer cells, how this is linked to metabolic deregulation in cancer, and may even lead to novel therapeutic vulnerabilities.
The presented findings are largely supported by the provided data, although they remain largely descriptive. Nonetheless, the authors need to provide some additional controls to solidify their findings, specifically when using inhibitors, for instance of MYC. Likewise, it remains debatable if changes in mRNA levels over comparatively short perturbation time frames are key indicators of altered pathway activities. To this end, the authors should consider evaluating protein levels and gene expression data by GSEA.
Suggestions to authors:
1. If the glutamine-dependency in SG formation is largely cancer cell specific, is it related to altered KRAS mutational status? This was reported to impact on the glutamine addiction of cancer cells. Although the authors provide evidence of conservation in A549 and MCF7 cells, it would be interesting to consider this aspect in additional cancer cell models with and without KRAS mutations.

We thank the reviewer for bringing up the connection between KRAS mutations and metabolic output; we were interested in this and cited in the text papers linking the two. In the first version of the manuscript, we performed experiments on A549 cells which contain KRAS mutations and did not observe any significant change in response to the treatments between the cell types. To probe the issue further as suggested, we also tested HCT116 colorectal cells that contain a KRAS mutation, and found that these also followed the same pattern with regard to glutamine supplement and subsequent SG formation. We have added these cells to supplemental figure S3.

2. In respect to the energy dependency, it would be informative to evaluate if other stressors

© 2025. Published by The Company of Biologists under the terms of the Creative Commons Attribution License (https://creativecommons.org/licenses/by/4.0/).

leading to specific ATP depletion, for instance by the FDA-approved drugs targeting ATP5F1C recapitulates the presented observations.

This was a welcomed suggestion by the reviewer. We employed the drug suggested - Oligomycin A - and indeed we saw a negation of the glutamine recovery. This was a positive reinforcement of the principle that both metabolic inputs of glutamine and glucose were essential for SG formation under starvation conditions. By inhibiting ATP synthase, ATP from the TCA cycle supported by glutamine was not obtained, and SGs were absent from all cells. We found this result very impactful and added to Figure 5A. The rest of the treatment conditions can be found below in Figure 1 for the reviewers.

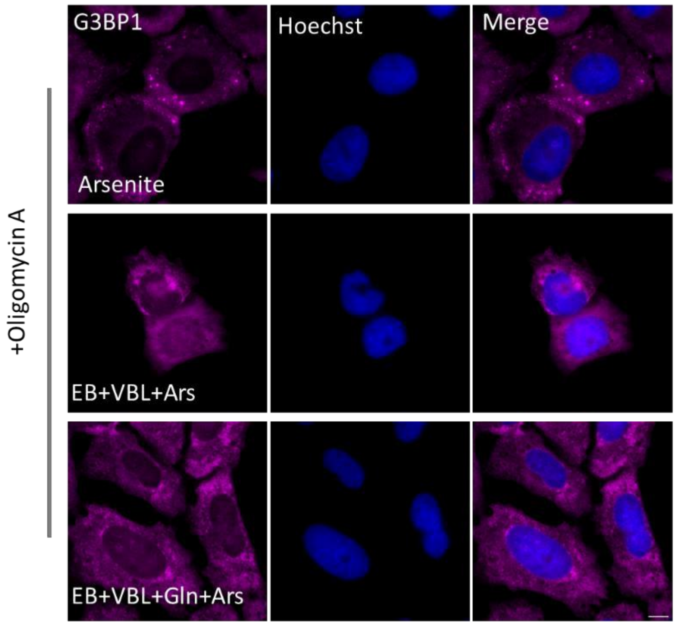

Figure 1 for reviewers: Inhibiting ATP synthase negates effect of glutamine recovery on SG formation. U2OS cells were AA-starved (EBSS, EB) + VBL, along with glutamine and then arsenite, followed by immunofluorescence staining with anti-G3BP1 as a SG marker (magenta). Oligomycin A was added to the cell culture as an ATP inhibitors and completely inhibited SG formation in glutamine recovered cells. Hoechst 33342 DNA stain is shown in blue. Scale bar, 10 µm.

3. The analyses in Fig.4 certainly support the authors conclusion that SGs operate in a larger context, in particular energy production. However, arsenite likely induces a largely energy-related stress affecting ATP production via mitochondria, as indicated also indicated in the manuscript. Thus, the authors should consider testing other stress inductions, e.g. ER, heat and/or hypoxic stress, to test if glutamine/Glu are equally important in such scenarios.

The reviewer observantly notes that arsenite has its own effects on energy pathways, therefore it was essential to test whether glutamine and amino acid starvation (EBSS) had the same general affects on cells exposed to other stressors. We therefore tested another type of oxidative stress, H_2O_2, and indeed saw that the cells followed the same pattern. We also administered a suggested ER stressor, thapsigargin, and saw that the SGs followed the energy principles observed with oxidative stress. These treatments were added as supplemental figure S5.

4. In respect to gene expression analyses, the authors should deepen their evaluation of NGS data by GSEA analyses to get more insights on altered pathway activities, e.g. MYC-driven transcription etc.

We thank the reviewer for suggesting this powerful NGS analysis. When comparing gene expression patterns between the glutamine recovered group versus that of starvation, there were expected hits for increased metabolism related pathways, and nucleic acid processing, as we had seen in the previous analyses (Figure S4A and Figure 2 for reviewers). However, what was a welcomed surprise was that the second most enriched pathway included ‘MYC targets V1’, which was of note, given

© 2025. Published by The Company of Biologists under the terms of the Creative Commons Attribution License (https://creativecommons.org/licenses/by/4.0/).

the importance of MYC in the manuscript. We had previously observed that MYC was one of the most upregulated genes after treatment, in both the starved and glutamine recovered group. But in this case, it was a special subsection of MYC targets that was upregulated after glutamine was administered to the media.

Upon further examination of the proteins involved, many of the proteins in this MYC mediated pathway actually modulate the stress response to oxidative stress (through L13a- ceruloplasmin silencing). This is of note because we demonstrated that the glutamine was essential for the cells to mount a proper stress response. Additionally, we saw protein clusters dealing with cell cycle, splicing, and mRNA processing, which we had encountered in the original NGS analysis. We validated these findings by examining nascent mRNA and DNA (data below, comment 5), and indeed observed a significant increase in both nascent transcription and DNA replication foci after glutamine addition.

Interestingly, when we examined which genes were elevated in the starved condition compared to the group that received glutamine, we also saw an elevated gene pathway for MYC targets, but of a different subset, termed ‘MYC targets V2’. This set of MYC targets dealt with almost exclusively rRNA and ribosomal subunits. This was likely due to the extreme lack of translational output (observed also with nascent protein imaging, data below), and this is a specific mechanism for the cell to address this concern. This was interesting to us because MYC was one of the most heavily upregulated genes in both the starved and recovered condition compared to cells grown in full media, but actually MYC in each case seems to have a slightly altered role, and the cell can modulate which set of MYC targets should be addressed given the metabolic state. We have also added the GSEA analysis to the manuscript. The enrichment plots were added as Figure S4E.

5. The findings of altered glutamine transport capacities require further validation. The authors should validate their findings on the protein level, since an adaption of mRNA levels may not properly reflect changes of transport capacity at the plasma membrane. Furthermore, how do the authors envision that stress induction promotes such changes in gene expression - at the transcriptional level or altered RNA turnover? Overall translation should be largely silenced in stressed cells?

This is an important point, and we fully agree that the gene expression data had to be validated on a protein level. Indeed, western blots show increased protein expression of SLC1A5 (primary glutamine transporter) concomitant to gene expression in both of the starved groups (EBSS), with and without glutamine addition. Because the starvation (EBSS) with or without glutamine is administered for ~18 hours, that is enough time for the cell to change its transport protein profile. This is why we observe such dramatic changes between the groups. We do observe a significant increase in nucleotide metabolism, made possible from nitrogen donated by the glutamine, which resulted in increased mRNA transcription and increased gene expression related to mRNA processing and DNA replication (Figure 2 for the reviewers). This certainly contributes the cell's overall increased viability. However, even in the starved (EBSS)+VBL treated group, we still observe transcription and translation, and the limited resources and energy available are devoted to increasing glutamine uptake. All the relevant data has been added to the manuscript in figure S6.

© 2025. Published by The Company of Biologists under the terms of the Creative Commons Attribution License (https://creativecommons.org/licenses/by/4.0/).

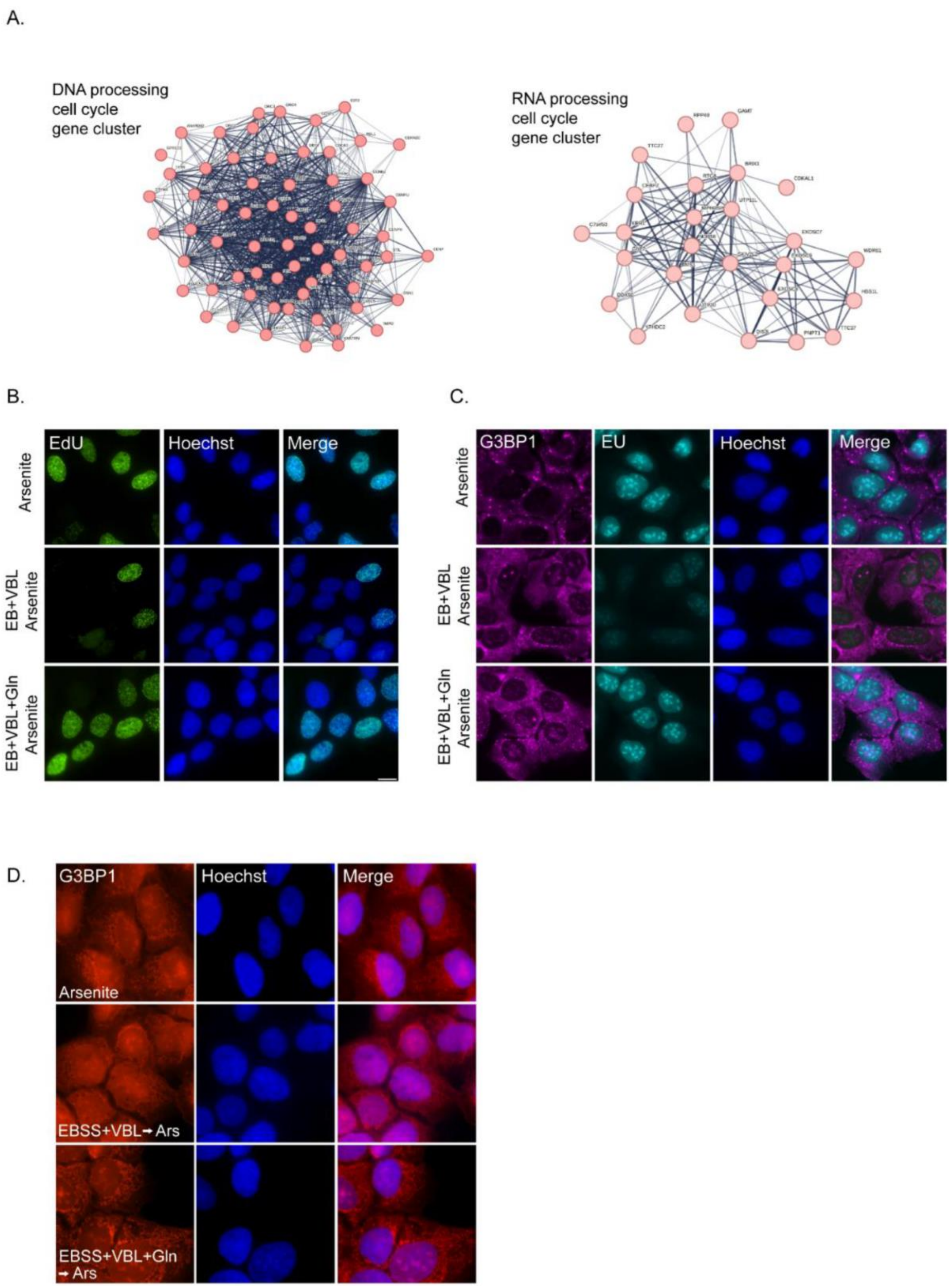

Figure 2 for the reviewers: Altered cellular processes following starvation and glutamine rescue: (A)
STRING network clusters for both DNA and RNA processing enriched in glutamine recovered cells. (B)
Nascent DNA replication foci were detected in U2OS cells using EdU staining (green). EBSS (EB) + VBL treated cells show significantly less replication foci compared to cells grown in full medium or those supplemented with glutamine. Hoechst 33342 DNA stain is shown in blue. Scale bar, 20 µm. (C) U2OS cells stained by immunofluorescence with anti-G3BP1 as a SG marker (magenta), along with detection of nascent RNA by EU (cyan). EBSS+VBL treated cells showed significantly less nascent RNA compared to cells grown in full medium or those supplemented with glutamine, likely contributing to the limited number of SGs after arsenite exposure. Scale bar, 20 µm. (D) Nascent protein imaged with HPG modified methionine. Nascent protein can be seen being traffic in and around the endoplasmic reticulum. While arsenite and glutamine recovered groups show significant

© 2025. Published by The Company of Biologists under the terms of the Creative Commons Attribution License (https://creativecommons.org/licenses/by/4.0/).

nascent protein, EBSS+VBL treated cells show limited nascent protein.

6. This referee is not an expert in 2DG-directed block of glycolysis and glycolysis-dependent ATP production. How can the authors exclude that 2DG also affect mitochondrial production of ATP and how can they potentially solidify their findings by additional controls or alternative inhibitors.

We appreciate this question. We believe that we are able to disentangle the two ATP measurements thanks to the Seahorse experiments performed. When the 2DG was added to the cells, there was a reduction in ATP production exclusively from the glycolytic fraction. This is an important question, because bulk ATP measurements would not allow for isolating of each element, but we can observe with the Seahorse that the 2DG indeed only effected the glycolytic fraction, while the glutamine was still able to support the mitochondrial fraction.

7+8. The findings on MYC are interesting. However, it remains to be clarified if changes in MYC act mainly via transcriptional regulation, for instance by monitoring MYC transcriptional reporter activity. In addition, it remains unclear if changes in MYC abundance are also seen on the protein level and if they result from altered transcription, mRNA or protein turnover. This should be addressed in view of the rather short perturbation times. Finally, the authors should control for MYCi and should investigate their gene expression data (Fig.4) for alterations in MYC pathways, for instance via GSEA studies (see above).

The observations in G3BP1/2-KO cells are interesting but would benefit from further in-depth testing of the MYC connection and altered gene expression levels. To this end, the authors should consider evaluating gene expression profiles by NGS and/or monitor MYC abundance at the protein level and its transcriptional activity by luciferase reporters.

We welcomed the idea of the MYC luciferase reporter in order to better understand MYC activity. We performed a luciferase assay with a reporter plasmid that contains 4 E-box sequences, the canonical MYC binding sites, to measure the levels of transcriptional activation under the various conditions. Significantly increased levels of MYC activity were found under the conditions that had more MYC gene expression.

In the knock out cells, there was no significant increase in the MYC transcriptional activity from arsenite exposed cells to those treated with amino acid starvation (EBSS)+VBL, and also no significant increase after the addition of glutamine. While WT and DKO cells grown in full media showed identical activity, the EBSS+VBL exposed groups showed significantly lower levels of MYC activity. We believe this is an important experimental validation of our suspected decrease in MYC activity observed in DKO cells, and their accompanied change in metabolic profile. The relevant information has been added to the text and the data are shown in Fig. S4D.

The reviewer correctly notes the necessity of MYC protein expression to compliment the gene expression data. Indeed, cells that showed highly elevated MYC gene expression also have concomitant increase in protein expression. While cells in full media showed about 1.2x MYC expression after arsenite exposure compared to untreated cells, the conditions in EBSS media showed around 1.7x (added to figure S6). As mentioned above, we believe that the cells with increased transcription due to available ATP after glutamine enable increased expression of MYC target transcripts . While the changes in gene expression of MYC itself are more dramatic than the changes at the protein level, we believe the cells are investing precious resources into transcription of the gene in order to fill all possible translation capacity. While it is clear that MYC activity and associated pathways are heavily enhanced after starvation, it is possible smaller changes in overall protein are sufficient to drive the effect. As expected, ΔΔG3BP1/2 cells showed a flattened level of MYC expression, where starvation did not stimulate any real change in MYC expression. This was expected, because ΔΔG3BP1/2 cells did not increase viability in a profound way after glutamine supplementation.

9. Minor comment: The investigated glutamine transporters in Fig.5C do not cover all glutamine transporters reported in mammalian cells, especially they lack SLC7A5 (LAT1), which is considered major to my knowledge. Could the authors comment on their selection and extend their analysis if appropriate?

© 2025. Published by The Company of Biologists under the terms of the Creative Commons Attribution License (https://creativecommons.org/licenses/by/4.0/).

We appreciate the reviewer for directing his attention to this point, because we had been interested in the idea that glutamine could act to increase the AA pool inside the cell, especially by acting as a substrate to aid transport for other amino acids. For this reason, we looked at the SLC7A5 and SLC3A2 antiporters, and saw increased expression levels in the groups that were starved. We postulated that because the cells were grown in media lacking AAs, they would overexpress genes that allowed for import/synthesis of amino acids. In the same vein we also saw an increase in the SLC7A11 antiporter, and a whole host of genes that link glutamine metabolism to AA synthesis (Figure 3 for the reviewers).

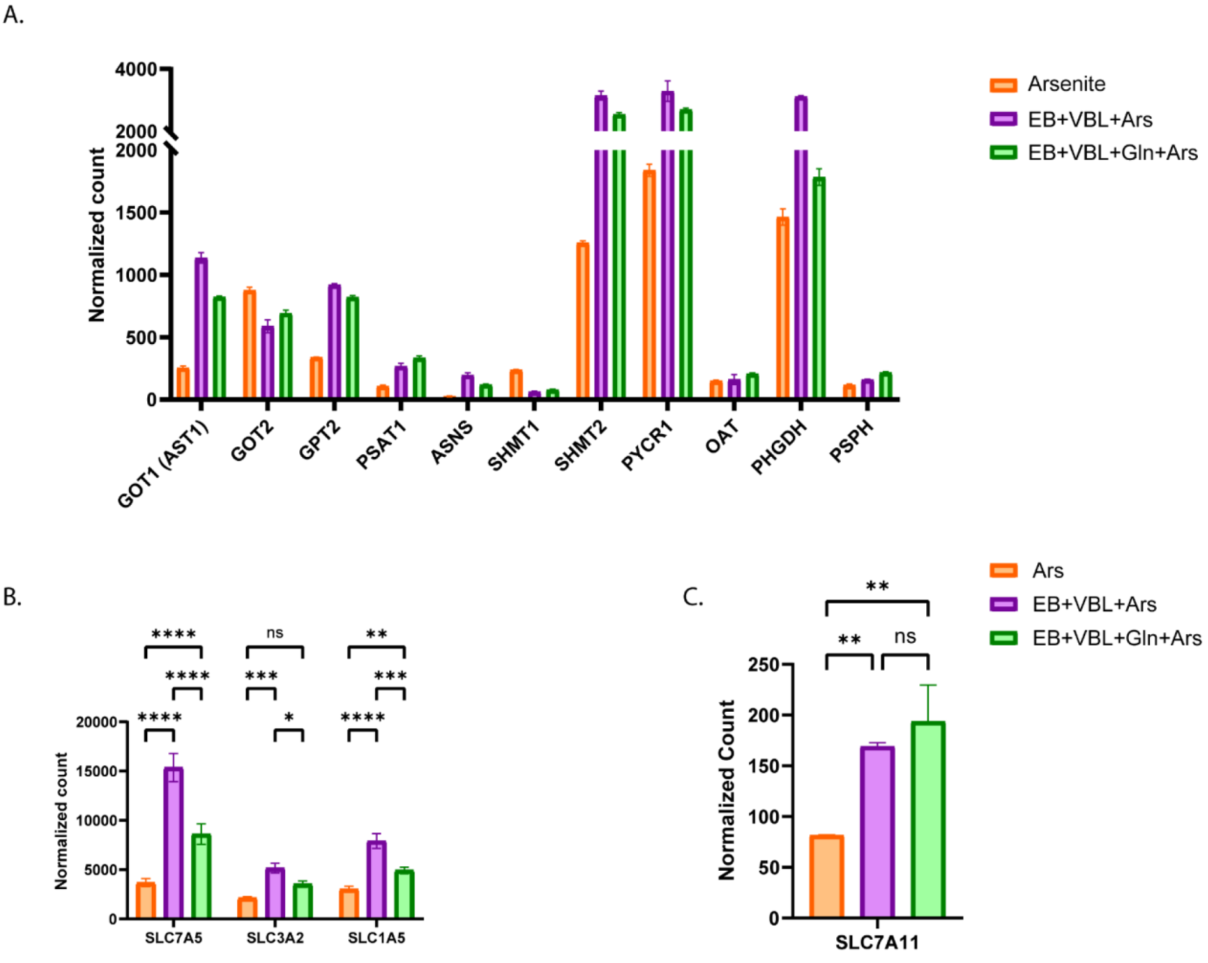

Figure 3 for the reviewers: Measuring gene expression levels of genes involved in metabolic mechanisms. (A) NGS normalized counts of multiple genes involved in AA synthesis. Most genes showed an increase after EBSS+VBL with and without glutamine, indicating cellular response to severe lack of available AAs. (B,C) NGS normalized counts of multiple cell transporters which use glutamine as a substrate to move AAs into the cell. There is an increase in transporter levels after EBSS+VBL with and without glutamine, demonstrating a cellular strategy to use glutamine to increase other AAs. (*P<0.05, **P<0.01, ***P<0.001, ****P<0.0001). Data was analyzed using one-way ANOVA.

Reviewer 2: SUMMARY OF THE ADVANCE MADE IN THIS PAPER AND ITS POTENTIAL SIGNIFICANCE TO THE FIELD
This is well-developed work. The results are interesting and novel. The finding that the sole amino acid source governing whether cancer cells form SGs during stress or not is of particular note. In agreement with it, metabolic profiling revealed the essential functions of glutamine and glucose in SG formation under limiting metabolic conditions. Interestingly, inhibition of MYC prevented SG formation, which is important finding of the work.
I only have few minor suggestions to improve the work.
SUGGESTIONS TO AUTHORS

© 2025. Published by The Company of Biologists under the terms of the Creative Commons Attribution License (https://creativecommons.org/licenses/by/4.0/).

1. Myc levels are determined on mRNA level. Authors should back up their myc mRNA changes by western blotting detecting Myc protein. I think the fact that Myc mRNA is found to be localized into SG may change its availability to translation and thus limit Myc protein production

We appreciate this comment, western blot quantifications are displayed above in comments 7/8. We suspect that not all MYC transcripts are sequestered in the granules, still, and we can observe changes in MYC protein expression related to our measured gene expression levels.

2+3. Core binding proteins. Authors should use siRNA to KD other "core" SG proteins that are influencing SG formation, e.g. UBAP2L and/or DDX6.

How does overexpression of G3BP affect SG behavior? Will it overcome metabolic "restrictions" for SG dynamics? Will it affect cell viability and survival?

This was an interesting point to investigate. We generated a DD6 KO cell line to observe how this would potentially effect SG formation and cell survival. Though these cells were lacking DD6, they still succeeded in forming SGs after arsenite exposure, and followed the same energy principles related to SG formation and therefore did not form SGs after AA starvation +VBL treatment (Figure 4 for the reviewers).

We also performed cell death assays on these cells to determine their viability after stress. Because SGs were detected in the glutamine recovered groups, we did not observe any noticeable change in cell death after treatment, compared to the WT groups.

Additionally, we quantified MYC with smFISH, and MYC levels appeared consistent with WT cells. Therefore, we conclude that not all significant proteins involved in SG formation affect cells in an identical manner. Data displayed together below in Figure 6 for the reviewers.

NOTE: an image provided in confidence for the reviewers has been removed

We also welcomed the interesting point of overexpression (OE). We generated a stable cell line expressing G3BP1-GFP. We observed that GBP1 OE was not capable of rescuing AA-starved+VBL treated cells. Similar to the experiments above, we also examined cell viability and MYC expression in these cells and saw no significant change. We conclude therefore that availability of G3BP1 is not a limiting factor in SG formation or metabolic profile changes after starvation and arsenite exposure (Figure 5 for the reviewers).

© 2025. Published by The Company of Biologists under the terms of the Creative Commons Attribution License (https://creativecommons.org/licenses/by/4.0/).

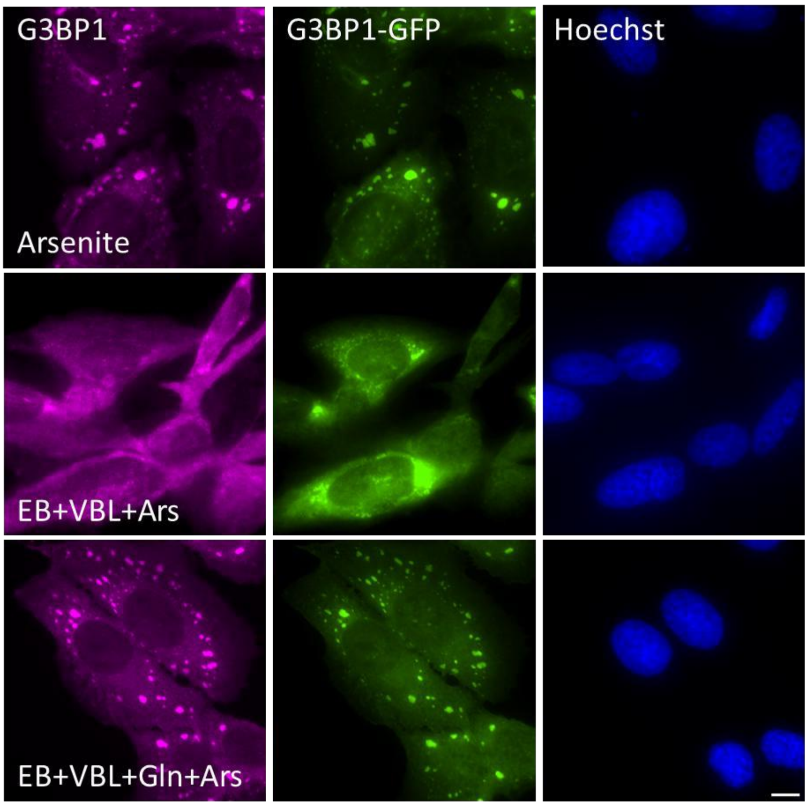

Figure 5 for the reviewers: Generation and treatment of cell line expressing G3BP1-GFP. U2OS cells stable expressing G3BP1-GFP were treated with aminoacid starvation (EB) + VBL along with glutamine and then arsenite, followed by immunofluorescence staining with anti-G3BP1 as a SG marker (magenta). While the cells followed the expected pattern of SG disappearance and return, G3BP1 OE did not dramatically affect SG formation. Hoechst 33342 DNA stain is shown in blue. Scale bar, 10 µm.

NOTE: an image provided in confidence for the reviewers has been removed

---

Second decision letter

MS ID#: jcs.263679R1

MS Title: Glutamine modulates stress granule formation in cancer cells through core RNA-binding proteins

Authors: Yaron Shav-Tal; Gabriel P Faber; Gross Gilad; Oz Mualem; Matan Y Avivi; Hiba Waldman Ben-Asher; Orly Yaron; Orit Shefi; Rakefet Ben-Yishay; Dana Ishay-Ronen

Article Type: Research Article

Dear Dr Shav-Tal,

I am happy to tell you that your manuscript has been accepted for publication in Journal of Cell Science, pending standard publication integrity checks.

© 2025. Published by The Company of Biologists under the terms of the Creative Commons Attribution License (https://creativecommons.org/licenses/by/4.0/).
